# Dietary Patterns, Food-Group Profiles, and Nutritional Characteristics in a Diverse Cohort of Young Adults Attending a UK University (2016)

**DOI:** 10.3390/nu18213503

**Published:** 2026-10-25

**Authors:** Antonio Peña-Fernández, Tomás Cámara-Pastor, M. Ángeles Peña Fernández

**Affiliations:** 1 Area of Legal and Forensic Medicine, Department of Surgery, Medical and Social Sciences, Faculty of Medicine and Health Sciences, University of Alcalá, Ctra. Madrid-Barcelona, Km. 33.6, 28871 Alcalá de Henares, Spain; 2 Leicester School of Allied Health Sciences, De Montfort University, Leicester LE1 9BH, UK; 3 Scientific Computation Research Institute (SCRIUR), University of La Rioja, 26006 Logroño, Spain; 4 Department of Biomedical Sciences, Faculty of Pharmacy, University of Alcalá, Crta. Madrid-Barcelona Km. 33.6, 28871 Alcalá de Henares, Spain

**Keywords:** dietary patterns, food groups, young adults, university students, food-frequency questionnaire, principal component analysis, nutritional epidemiology, geographical background, United Kingdom

## Abstract

Background/Objectives: Whole-diet patterns in geographically diverse university populations remain insufficiently characterised. We examined food-group consumption and dietary patterns according to sex and geographical background in a historical UK university cohort. Methods: This cross-sectional analysis included 111 students assessed at De Montfort University, Leicester, in early 2016. Habitual intake was assessed using an expanded, adapted EPIC-Norfolk food-frequency questionnaire. Fifteen food groups were evaluated using principal component analysis after log1p transformation and standardisation; between-group comparisons were adjusted for relevant covariates and subjected to Benjamini–Hochberg false-discovery-rate correction. Results: The participants had a mean age of 20.4 ± 1.2 years, and 70.3% were female. Three patterns—Plant-rich, Animal-source foods, and Energy-dense/discretionary—explained 46.25% of the variance, compared with 35.24% for the two-component alternative. Parallel analysis supported retention of three components; bootstrap results favoured their overall reproducibility but indicated substantial uncertainty in individual food-group loadings. Males had lower adjusted Plant-rich scores than females (−0.43 SD, 95% CI −0.76 to −0.10; *q* = 0.04). Plant-rich and Animal-source foods scores differed by geographical background (*q* = 0.006 for both global tests), whereas the Energy-dense/discretionary pattern did not. Conclusions: These exploratory patterns characterise dietary heterogeneity in this historical cohort but require replication in larger, contemporary samples.

## 1. Introduction

Young adulthood represents a major developmental transition during which individuals progressively acquire greater autonomy over food purchasing, preparation, meal timing, social eating, and other health-related behaviours [1]. For many young adults, entry into higher education intensifies this transition through changes in residence, financial circumstances, schedules, social networks, access to cooking facilities, and exposure to new food environments [2,3]. These changes may favour both positive and unfavourable dietary behaviours and can occur at a stage when behavioural patterns with potential long-term consequences are being established [1,2,3].

University populations therefore represent an important group for nutritional research. Studies in British university students have documented considerable heterogeneity in eating behaviour, including frequent consumption of snack and convenience foods alongside variable fruit and vegetable intake [4]. Nutrition knowledge also differs substantially among UK university students and does not necessarily translate directly into healthier food choices, emphasising that eating behaviour arises from an interaction between knowledge, individual preferences, resources, social context, and the surrounding food environment [5]. Recent international evidence similarly indicates that poor diet quality in university populations is associated with intersecting demographic, behavioural, academic, and contextual characteristics [6], while food choice may differ according to dietary identity, eating setting, and the motivations attached to specific food categories [7].

Food-group consumption provides a complementary perspective to total energy and nutrient intake. The relative consumption of fruit, vegetables, cereal products, animal-source foods, discretionary snacks, and beverages can influence the overall nutritional composition of the diet. University students with similar total energy intakes may therefore exhibit substantially different food-group profiles, highlighting the importance of assessing both individual food groups and their combinations.

An important consequence is that university diets cannot be adequately characterised by examining isolated nutrients alone. Foods and nutrients are consumed as combinations, and intake of one dietary component is frequently correlated with intake of others. Dietary-pattern analysis has therefore emerged as a complementary approach in nutritional epidemiology because it evaluates the overall structure of food consumption rather than treating each nutrient or food as an independent exposure [8,9]. Empirically derived approaches, including principal component analysis (PCA), can identify correlated dietary dimensions from observed food intake and provide continuous pattern scores representing the extent to which each individual expresses those dimensions [8,9].

This approach has previously been applied to UK university populations. In a cross-sectional study of 1448 students attending five UK universities, Sprake et al. identified four major patterns described as vegetarian, snacking, health-conscious, and convenience, red meat and alcohol [10]. Pattern adherence differed according to sex and several lifestyle and sociodemographic characteristics, demonstrating substantial heterogeneity within the broad category of “university students” [10]. However, empirically derived dietary patterns are inherently population- and method-dependent. Their structure may be influenced by the foods assessed, grouping decisions, cultural composition of the study population, statistical transformation, component-retention criteria, and the wider food environment [8,9,10]. Independent characterisation in different university settings is therefore informative rather than simply replicative.

Such heterogeneity may be particularly relevant in universities serving students from diverse cultural and geographical backgrounds. Food practices may be influenced by family traditions, religion, migration and acculturation, food availability, income, cooking skills, living arrangements, and individual identity [2,7]. Broad geographical categories cannot capture this complexity and should not be interpreted as equivalent to ethnicity or as intrinsic determinants of dietary behaviour. Nevertheless, when used cautiously, they can identify heterogeneity that warrants further investigation and may help prevent the assumption that a single dietary profile characterises an entire student population.

UK dietary policy likewise emphasises dietary patterns rather than individual nutrients in isolation. The Eatwell Guide promotes a balanced dietary structure centred on fruit and vegetables, higher-fibre starchy carbohydrates, protein foods, and dairy or alternatives, while limiting foods high in fat, salt, and sugars [11]. The Scientific Advisory Committee on Nutrition (SACN) recommends an average population dietary fibre intake of approximately 30 g/day for individuals aged 16 years and over [12]. The National Diet and Nutrition Survey (NDNS) provides the principal nationally representative framework for monitoring food consumption and nutrient intake in the UK [13,14]. Nevertheless, national surveillance cannot fully describe the more specific dietary environments encountered within individual university populations.

The present study addresses this gap through a de novo reconstruction and whole-diet analysis of dietary data collected from young adults attending De Montfort University (DMU), Leicester, UK. The cohort is of particular interest because it includes substantial representation from African, Asian, and European geographical backgrounds and combines detailed dietary information with measured anthropometric and body-composition characteristics.

As participant assessments were conducted in early 2016, the present investigation provides a historical characterisation of dietary behaviour rather than an estimate of current dietary practices among UK university students. The availability of detailed food-frequency data, measured anthropometry, and bioelectrical impedance-derived body-composition estimates obtained under a common study protocol provides an opportunity to examine dietary heterogeneity within this cohort. These findings may also serve as a historical reference for future investigations, although changes in university food environments and dietary practices over the subsequent decade must be considered when interpreting their broader applicability.

The primary objective of this study was to characterise habitual food-group consumption and identify empirically derived dietary patterns using PCA in a geographically diverse cohort of young adults attending a UK university. The secondary objective was to investigate differences in dietary-pattern scores and individual food-group intakes according to sex and geographical background using appropriately adjusted statistical models. The present analysis focused on whole-diet structure and food-group heterogeneity, while detailed nutrient-adequacy assessment and diet–adiposity associations were reserved for separate investigations.

## 2. Materials and Methods

### 2.1. Study Design, Setting, and Recruitment

This historical cross-sectional study represents the nutritional component of a wider investigation initiated during the 2015/2016 academic year at De Montfort University (DMU), Leicester, UK [15]. Study planning and preparation of participant-facing materials began in late 2015, whereas participant recruitment and data collection contributing to the present analysis were undertaken following ethical approval in early 2016. Data collection was concentrated between January and mid-March 2016 and completed before Easter vacation. The present analysis was restricted to dietary, anthropometric, body-composition, blood-pressure, and pulse data retained in the final validated nutritional dataset.

Eligible university students were recruited through complementary routes across DMU’s four faculties: Art, Design and Humanities; Business and Law; Health and Life Sciences; and Technology. The study was advertised through brief presentations during scheduled lectures, including presentations towards the end of teaching sessions; announcements in the Blackboard virtual learning environments of participating modules; information posters and other study materials displayed within the university; and dissemination through student/course representatives [15]. Because these approaches overlapped and could expose the same student to the study on more than one occasion, a reliable denominator for all students who encountered recruitment information was not available and a formal response rate was therefore not calculated.

Interested students received written study information and were offered flexible face-to-face appointments to facilitate participation. Under an approved amendment to the study protocol, participants received a £5 voucher in recognition of the time required to complete the study procedures. Participation remained voluntary, and written informed consent was obtained before data collection.

Study assessments were conducted by appointment at the Biomedical and Environmental Health Research Laboratory, Leicester School of Allied Health Sciences, De Montfort University, Leicester, UK. Participants completed the dietary questionnaire and underwent the measurements included in the present analysis. Assistance with questionnaire completion was available from a member of the research team when required.

Eligibility for the university component of the parent study comprised enrolment at DMU, age 18–22 years, general good health, and written informed consent. The present nutritional analysis included participants with identifiable dietary records containing sufficient and interpretable food-frequency and portion-size information to generate quantitative intake estimates. The final quality-controlled dataset comprised 111 participants. No additional clinical exclusion criteria or retrospective numerical FFQ-completion thresholds were applied.

Recruitment was conducted through several overlapping university-wide communication channels, and a unique denominator of students invited to participate was not available. The retained historical records also do not permit reliable reconstruction of the numbers screened or excluded before the final analytical dataset was established. Consequently, a formal response rate or complete participant-flow diagram could not be calculated.

Geographical background was derived from the recorded continent of birth and was grouped as Africa, Asia, Europe, or Other. This variable was used as a broad geographical descriptor and was not treated as a proxy for ethnicity, culture, religion, or migration history. The Other category comprised two participants and was retained in descriptive summaries but excluded from formal geographical-background comparisons because of the insufficient group size.

Formal ethical approval was granted by Chair’s Action by the Faculty Research Ethics Committee, Faculty of Health and Life Sciences, De Montfort University (Ref. 1674), on 11 January 2016. Participant recruitment, informed consent, and the study procedures contributing data to the present analysis were undertaken following ethical approval. Subsequent protocol amendments were approved through the institutional ethics process. The study was conducted in accordance with the Declaration of Helsinki. Recruitment procedures for the wider project have been described previously [15].

### 2.2. Anthropometric, Body-Composition, and Physiological Measurements

Age and sex were recorded for each participant. Standing height was measured during the study appointment by an NHS-registered nurse using a seca 213 portable stadiometer (seca GmbH & Co. KG, Hamburg, Germany), with 1 mm graduation. Body weight and body-composition estimates were obtained using a Tanita SC-330-S body-composition analyser (Tanita, London, UK), based on bioelectrical impedance analysis (BIA) [16,17]. The Tanita SC-330-S has been used for anthropometric and body-composition assessment in previous research involving young university students [16].

To standardise premeasurement conditions, participants were instructed before their appointment to refrain from food intake, alcoholic beverages, coffee and other caffeinated beverages, smoking, and physical exercise for at least 8 h before assessment; only water was permitted during this period. These procedures were intended to minimise short-term variation in bioimpedance associated with recent food or beverage consumption and physical activity [17]. Body-composition measurements were performed barefoot in accordance with the manufacturer’s operating procedure.

Body-composition estimates comprised body fat (%), total body water (%), muscle mass (kg), bone mass (kg), visceral-fat index, and basal metabolic rate (BMR; kcal/day). Systolic and diastolic blood pressure were assessed during the same study appointment using an OMRON M6 automated upper-arm blood-pressure monitor (HEM-7001-E; OMRON Healthcare, Kyoto, Japan). The device uses the oscillometric principle and fulfills the validation criteria of the International Protocol of the European Society of Hypertension [18]. Three repeated blood-pressure measurements were obtained for each participant, and their arithmetic mean was used as the participant-level analytical value for systolic and diastolic blood pressure (mmHg). Pulse rate (beats/min) was recorded using the same device, and the quality-controlled participant-level value retained in the study database was used for analysis.

Body mass index (BMI) was recalculated from the validated height and body-weight measurements as

BMI (kg/m^2^) = body weight (kg)/height^2^ (m^2^).

For descriptive purposes, conventional adult BMI categories were applied: underweight, <18.5 kg/m^2^; normal weight, 18.5–24.9 kg/m^2^; overweight, 25.0–29.9 kg/m^2^; and obesity, ≥30.0 kg/m^2^.

Anthropometric and body-composition data were reconciled against the original study records before analysis. Raw source variables and derived variables were retained separately, and all subsequent analyses used the validated participant-level dataset.

### 2.3. Dietary Assessment

Habitual dietary intake was assessed using an expanded semi-quantitative food-frequency questionnaire (FFQ) developed for the parent study. Its core structure was based on an adapted version of the EPIC-Norfolk FFQ, version 6 (CAMB/PQ/6/1205), whose underlying nutritional methodology has been described previously [19]. Questionnaire development also drew on the research group’s previous experience using a semi-quantitative FFQ to estimate dietary intake among young Spanish adults [20]. The expanded questionnaire administered in the present cohort was not independently validated against a reference dietary-assessment method.

The administered questionnaire contained more than 130 food and beverage items. Unlike the original EPIC-Norfolk FFQ, which was designed to estimate habitual intake over the preceding year [21], the adapted instrument used in the present study assessed food and beverage consumption over approximately the preceding four weeks. For each listed food or beverage, participants selected one of nine consumption-frequency categories, ranging from no consumption to six or more occasions per day. Portion sizes were recorded as small, medium, or large relative to predefined food-specific reference servings. Where appropriate, reference servings were expressed using familiar household measures or units, including slices, cups, glasses, and teaspoons. The expanded food list was designed to improve coverage of foods consumed within a geographically diverse UK university population. Examples included chapatti, naan, bazlama, mantou, couscous, plantain, yam, black-eyed beans, adzuki beans, curries, stir-fried noodles, fried rice, Pad Thai, tofu, and tempeh. These foods were explicitly represented in the questionnaire rather than assigned according to participants’ geographical background.

In addition to the food-frequency tables, the questionnaire included supplementary questions on usual milk consumption, types of fats used in food preparation, selected dietary restrictions, fruit-juice fortification, and nutritional supplement use. These questions broadened the dietary information collected but were not treated as separate input variables in the 15-food-group PCA. The analytical food-group assignments were subsequently established from the original food-level records, as detailed in Section 2.4 and Section 2.5.

Completed FFQs were processed using Nutritics^®^ Research Edition, version 5.7 (Nutritics Ltd., Dublin, Ireland). Food-composition interpretation was informed by established UK food-composition resources, including McCance and Widdowson’s *The Composition of Foods* [22]. Food-level names and original software categories were retained within the Nutritics/Access source database to enable subsequent auditing of dietary entries and their analytical classification. Earlier reports from this cohort addressed selected nutrient-related questions [23,24,25]; the present investigation reconstructed food-group intake and dietary patterns from participant-level data using a separate, reproducible analytical workflow.

### 2.4. Reconstruction and Quality Control of Dietary Data

Dietary data were reconstructed at participant level from the original Nutritics/Access database. Food records were subsequently standardised, aggregated, and mapped to analytical food groups as described below.

The source variable containing food weight, labelled (g), represented weekly intake. Daily intake was therefore calculated as g/day = g/week ÷ 7. This conversion was applied only to dietary variables recorded on a weekly basis; all demographic, anthropometric, physiological, and other non-dietary variables were retained in their original units. Dietary food weights and nutritional summary variables were checked separately to preserve their original unit definitions. Nutritional variables already available in the validated analytical dataset as daily intake estimates were not subjected to an additional division by seven. This distinction was maintained throughout the reconstruction and quality-control workflow to avoid duplicate unit conversions. Minimal text normalisation reduced the 78 raw Category2 labels to 77 semantically distinct categories without altering the underlying source classifications. Original category labels were retained to preserve data provenance.

Multiple records corresponding to the same participant, *FoodName*, and source category were retained when they represented distinct recipe or food-component contributions and were summed during aggregation. Participant–category combinations not represented in the positive-only source table were treated as structural zeros. Aggregated participant totals were verified against the source data before analysis.

The complete analytical workflow is shown in Figure S1.

### 2.5. Derivation of Analytical Food Groups

The 77 normalised categories were mapped to 18 analytical food groups using an audited, reproducible mapping procedure. Original *FoodName*, raw Category2, normalised Category2, historical grouping, and final analytical grouping were retained, permitting the derivation to be traced back to the participant-level source.

Classification decisions were based on the original food-level descriptions, culinary identity, and intended analytical role of each food, rather than relying exclusively on the broad categories assigned by the dietary software. Cereals/Grains principally represented staple cereal products and grain-based foods, whereas Discretionary foods included sweet biscuits, confectionery, and other predominantly snack-type products. Accordingly, biscuits were allocated to Discretionary foods, while the Pastries and Buns category was divided at *FoodName* level to distinguish cereal-based bakery products from discretionary sweet products.

Pizza was retained within Cereals/Grains in the original analytical mapping, consistent with the inclusion of pizzas within the cereal-products classification of UK food-composition resources. This grouping was an analytical convention and should not be interpreted as indicating that pizza is nutritionally equivalent to plain cereal products. The original source category “Recipe” was traced to a single distinct food entry, “Porridge Builder”, which was allocated to Cereals/Grains on the basis of its porridge designation. The precise ingredient composition of this customised recipe could not be independently reconstructed. These classifications were retained consistently with the original food-group matrix used for the present principal component analysis (PCA; see Section 2.6).

The analytical food groups were designed to represent interpretable dimensions of habitual food consumption while limiting the number of variables relative to the available sample size. Three groups—Water, Mixed dishes/Condiments, and Other/Non-pattern—were retained in the reconstructed dataset but excluded as separate variables from the PCA. Water was excluded because its consumption primarily reflects hydration and could disproportionately influence analyses based on food weight rather than nutritional composition. Mixed dishes/Condiments combined heterogeneous products, including soups, sauces, condiments, and composite preparations, whose nutritional characteristics could not be represented reliably by a single, consistent food-group construct. Other/Non-pattern included miscellaneous food additives, supplements, and products that did not form a sufficiently interpretable dietary exposure.

These exclusions were methodological decisions intended to improve interpretability, not assumptions that the excluded foods were nutritionally unimportant. Their contributions to reported nutrient intake remained part of the original dietary assessment. Nevertheless, excluding heterogeneous mixed dishes from the PCA may limit representation of some food-consumption behaviours, particularly where composite meals are important components of habitual diets. The resulting patterns should therefore be interpreted as dimensions derived from the 15 selected analytical food groups rather than an exhaustive representation of every recorded food item.

The final 15 PCA candidate groups were as follows: Alcoholic beverages; Cereals/Grains; Dairy/Alternatives; Discretionary foods; Eggs; Fats/Oils; Fish/Seafood; Fruit; Nuts/Seeds; Poultry; Pulses; Red/processed meat; Soft/energy/sports drinks; Tea/Coffee; and Vegetables.

*FoodName*-level allocation rules were applied where the original software category did not adequately describe the analytical food group. These rules were incorporated into the derived mapping used to construct the final food-group matrix, while preserving the original software classifications and food-level descriptions for auditing. The retained PCA was based on the resulting fixed matrix of 111 participants and 15 analytical food groups.

The complete mapping of the 77 normalised source categories to the 18 analytical food groups, including identification of the 15 groups retained for PCA, is provided in Supplementary Table S1.

### 2.6. Dietary-Pattern Analysis

PCA was used to identify correlated dimensions of food-group consumption [8,9,10,26,27,28]. Analyses were based on the 15 predefined food groups rather than on the complete nutrient panel, thereby focusing the analysis on patterns of food consumption.

Food-group intake distributions contained structural zeros and pronounced right-skewness. Daily food-group intakes were therefore transformed as log1p(g/day) = ln[1 + intake (g/day)] and subsequently standardised to a mean of 0 and SD of 1 before the primary PCA. The corresponding untransformed g/day matrix was retained as a sensitivity analysis. Sampling adequacy and matrix suitability were evaluated using the Kaiser–Meyer–Olkin (KMO) statistic, individual KMO values, Bartlett’s test of sphericity, and inspection of the food-group correlation matrix.

PCA was implemented using stats::prcomp() in R. Component retention incorporated parallel analysis, scree/eigenvalue inspection, loading structure, communalities, nutritional interpretability, sensitivity to analytical scale, and bootstrap stability [26,27,28]. The final parallel analysis used 1000 simulated datasets. For each simulation, eigenvalues were obtained from a random matrix of the same dimensions as the observed dataset, and the observed eigenvalues were compared with the corresponding 95th percentile of the simulated eigenvalue distributions. The procedure was implemented directly using functions from the R stats package (cor, eigen, rnorm, and quantile). Both two- and three-component solutions were fitted to the same matrix of 111 participants and 15 standardised log1p-transformed food groups, applying orthogonal varimax rotation with Kaiser normalisation to each solution. The complete rotated loading matrix, communalities, and variance explained for the alternative two-component solution are presented in Supplementary Table S2. Complementary PCA suitability and component-retention diagnostics are provided in Supplementary Table S3. The three-component solution was retained following joint consideration of parallel-analysis evidence, scree/eigenvalue structure, nutritional interpretability, and comparative bootstrap stability.

The retained three-component solution was subjected to orthogonal varimax rotation with Kaiser normalisation, implemented using stats::varimax() with normalize = TRUE. Participant dietary-pattern scores were calculated using regression-type scoring from the rotated loading matrix. Score coefficients were obtained as B = R^−1^L, where R is the correlation matrix of the standardised food-group variables and L is the varimax-rotated loading matrix; individual pattern scores were then calculated as Scores = XB, where X is the standardised log1p-transformed dietary matrix. Pattern scores were subsequently re-standardised to a mean of 0 and SD of 1.

Because component signs are mathematically arbitrary, component loadings and their corresponding participant scores were reoriented, where necessary, so that the principal food groups defining each pattern had positive loadings. This sign reorientation does not alter the statistical solution, variance explained, or relative ranking of participants. An oblique promax rotation (m = 4) was additionally examined as a rotation-sensitivity analysis; orthogonal varimax was retained for the primary solution.

Bootstrap stability was evaluated using 1000 participant-level bootstrap resamples for each of the candidate two- and three-component solutions. Participants were resampled with replacement, and the complete analytical sequence—log1p transformation, standardisation, PCA, and varimax rotation—was repeated within each bootstrap replicate. Bootstrap components were subsequently aligned with the corresponding reference solution by component permutation and sign orientation before the stability of the loading structure was evaluated. Component-level reproducibility was evaluated using correlations between each aligned bootstrap loading vector and its corresponding reference loading vector, summarised by median and fifth-percentile correlations. Individual food-group loading uncertainty was assessed using percentile-based 95% bootstrap intervals. These complementary measures were interpreted separately, since reproducibility of the overall component structure does not necessarily imply precise estimation of every individual loading.

Pattern labels were assigned after examination of the retained loading structure and were intentionally descriptive rather than imposed labels such as “Western” or “Prudent”.

### 2.7. Statistical Analysis

All final statistical analyses were performed in R version 4.4.2 (R Foundation for Statistical Computing, Vienna, Austria) [29]. PCA, varimax and promax rotations, parallel analysis, and bootstrap-stability procedures were implemented using base R and stats version 4.4.2. The parallel-analysis and bootstrap procedures were implemented directly rather than through dedicated external packages. ggplot2 version 4.0.3, patchwork version 1.3.2, and svglite version 2.2.1 were used exclusively for graphical preparation and export.

Continuous descriptive variables are reported using both mean (SD) and median [interquartile range (IQR)] where appropriate. Categorical variables are reported as *n* (%). Reporting of both arithmetic and distribution-resistant summaries was retained because several dietary variables showed substantial right-skewness.

For regression analyses, continuous anthropometric variables were standardised on their original measurement scale. Total energy intake, macronutrient intake, fibre intake, and food-group variables were log1p-transformed and subsequently standardised. Dietary-pattern scores were also standardised. Adjusted regression coefficients can therefore be interpreted as differences in outcome SD units.

Sex models were adjusted for age and geographical background. For dietary outcomes other than total energy intake, models were additionally adjusted for log-transformed total energy intake. Total energy intake itself was adjusted for age and geographical background but was not adjusted for energy.

Geographical-background models included sex and age as covariates. Dietary outcomes other than total energy intake additionally included log-transformed total energy intake. The analytical geographical-background groups were Africa, Asia, and Europe. The two participants classified as Other were retained in overall descriptive analyses but excluded from formal geographical-background inference because the cell size was insufficient for reliable group estimation. Formal geographical analyses therefore included *n* = 109.

Total-energy adjustment was used to distinguish differences in dietary composition from differences driven principally by greater or lower total intake [30]. Consequently, energy-adjusted coefficients should not be interpreted as direct estimates of absolute group intake.

Unadjusted sex differences were additionally summarised using Hedges’ *g* with 95% CIs [31]. Global geographical-background effects were quantified using partial η^2^, followed by model-based pairwise contrasts where appropriate.

Because multiple related outcomes were evaluated, the Benjamini–Hochberg procedure was used to control the false discovery rate (BH-FDR) [32]. Adjustment was performed within predefined analytical families: cohort anthropometry, core diet, food groups, and dietary-pattern scores. Both unadjusted *p*-values and FDR-adjusted *q*-values were retained in the complete outputs. Main-text interpretation prioritised effect estimates, 95% CIs, predefined global tests, and results remaining robust after BH-FDR correction rather than isolated nominal *p*-values.

Energy-intake plausibility was examined during quality control using the relationship between reported energy intake and BMR [33,34]. However, sufficiently complete participant-level physical-activity information was not available to assign defensible individual physical-activity-level values for a formal Goldberg classification. No participant was excluded solely on the basis of reported energy intake.

This study is reported in accordance with the principles of the STROBE statement for cross-sectional observational studies [35].

### 2.8. Generative AI and AI-Assisted Technologies

During manuscript preparation, ChatGPT (OpenAI; GPT-5.6) was used to assist with manuscript structuring, language drafting and refinement, and graphical presentation. It was not used to generate or modify study data, perform the statistical analyses, or independently determine the scientific interpretation. All AI-assisted outputs were critically reviewed, verified, and edited by the authors.

## 3. Results

### 3.1. Characteristics of the Study Population

The final analytical cohort comprised 111 young adults, including 78 females (70.3%) and 33 males (29.7%). Geographical background was classified as Africa for 41 participants (36.9%), Asia for 41 (36.9%), Europe for 27 (24.3%), and Other for 2 (1.8%) (Table 1).

Mean age was 20.4 ± 1.2 years, mean body weight was 66.4 ± 13.5 kg, and mean BMI was 23.7 ± 4.1 kg/m^2^. Based on conventional BMI categories, 64 participants (57.7%) were classified as normal weight, 10 (9.0%) as underweight, 28 (25.2%) as overweight, and 9 (8.1%) as having obesity.

When overweight and obesity were considered together, 37 participants (33.3%) had a BMI of at least 25 kg/m^2^. Although obesity alone was relatively uncommon, the cohort should not therefore be regarded as uniformly having a favourable weight status. Adjusted comparisons showed several sex-related differences in anthropometric and physiological variables, including height, body composition, and systolic blood pressure, whereas BMI differences did not remain statistically significant after BH-FDR correction. Global geographical-background comparisons also identified significant differences in selected anthropometric characteristics, including height, muscle mass, and bone mass (Supplementary Tables S4 and S5).

Mean body fat was 25.7 ± 10.4%, body water was 53.0 ± 6.7%, muscle mass was 46.4 ± 8.8 kg, and bone mass was 2.5 ± 0.4 kg. Mean systolic and diastolic blood pressures were 119.0 ± 13.7 and 69.7 ± 8.6 mmHg, respectively, and mean pulse rate was 75.0 ± 11.4 beats/min (Table 1).

### 3.2. Overall Dietary and Food-Group Profile

The overall dietary profile is shown in Table 2. Mean total energy intake was 2524.1 ± 1256.1 kcal/day, with a median of 2245.4 kcal/day [IQR 1664.1–3114.5]. Median protein, total fat, and carbohydrate intakes were 104.2 [76.1–140.0], 77.7 [54.6–107.0], and 268.2 [195.4–390.6] g/day, respectively.

Further characterisation of lipid and carbohydrate intake showed mean saturated fatty acid (SFA), monounsaturated fatty acid (MUFA), and polyunsaturated fatty acid (PUFA) intakes of 31.1 ± 16.9, 32.4 ± 16.2, and 13.4 ± 7.5 g/day, respectively. The corresponding median intakes were 26.9 [19.8–36.1], 28.2 [21.6–38.6], and 11.2 [8.3–16.6] g/day. Mean total sugar intake was 144.0 ± 91.7 g/day, with a median of 121.7 [83.4–160.4] g/day (Table 2). These variables are presented descriptively to provide additional nutritional context and were not included as separate inputs in the food-group PCA.

Median fibre intake was 25.4 g/day [16.8–37.0], whereas the corresponding arithmetic mean was 28.6 ± 15.5 g/day. The discrepancy between mean and median for several variables reflected the pronounced right-skewness of individual dietary intake distributions.

The substantial variability observed across several nutrient and food-group variables reflects heterogeneity in reported dietary consumption within the cohort. For positively skewed variables, the arithmetic mean was often higher than the median, indicating that relatively high reported intakes among some participants influenced the overall mean. This was particularly evident for selected beverage categories. Variability alone does not establish statistically significant between-group differences, which were evaluated separately using the adjusted models presented in the supplementary tables.

Among solid or semi-solid analytical food groups, some of the largest median daily intakes were observed for Cereals/Grains (291.9 g/day), Vegetables (277.4 g/day), Fruit (239.3 g/day), and Dairy/Alternatives (216.3 g/day). Median intake of Discretionary foods was 101.7 g/day, Red/processed meat 87.7 g/day, Poultry 79.3 g/day, Pulses 39.4 g/day, and Fish/Seafood 37.1 g/day.

Several beverage-related groups showed particularly heterogeneous distributions. Median Soft/energy/sports drink intake was 225.3 g/day [70.7–667.9], compared with an arithmetic mean of 439.3 ± 558.1 g/day. Median Tea/Coffee intake was 111.4 g/day [35.6–286.5]. Median intake of Alcoholic beverages was 0.0 g/day [0.0–4.0], consistent with a substantial proportion of non-consumers.

### 3.3. Suitability of the Food-Group Matrix and Identification of Dietary Patterns

The food-group matrix showed acceptable suitability for exploratory PCA. For the primary log1p-transformed matrix, overall KMO was 0.700, and the minimum individual KMO was 0.614. Bartlett’s test strongly rejected the null hypothesis of an identity correlation matrix (*p* < 0.001). The maximum absolute pairwise correlation was 0.610, providing no evidence of problematic near-collinearity among the 15 candidate food groups.

The transformation-sensitivity assessment showed a clear difference in component retention. Parallel analysis, based on 1000 simulated datasets and comparison with the corresponding 95th-percentile eigenvalue thresholds, supported one component for the untransformed g/day matrix and three components for the primary log1p-transformed matrix (Supplementary Table S3; Figure S2). In the transformed analysis, the third observed eigenvalue exceeded the corresponding simulated threshold, whereas the fourth did not, supporting retention of three components.

The alternative two-component solution explained 35.24% of total food-group variance, comprising 18.40% and 16.84% for the first and second components, respectively (Supplementary Table S2). The retained three-component solution explained 46.25%, representing an additional 11.01 percentage points. Although variance explained necessarily increases when additional components are retained, the three-component solution was also supported by parallel analysis, greater nutritional interpretability, and higher component-level bootstrap reproducibility.

Bootstrap assessment using 1000 participant-level resamples for each candidate solution indicated greater component-level reproducibility for the three-component solution. Median correlations between the reference and bootstrap loading vectors ranged from 0.858 to 0.888 for the three-component solution, compared with 0.749 to 0.776 for the two-component solution. The corresponding fifth-percentile correlations ranged from 0.450 to 0.586 and from 0.089 to 0.274, respectively.

Nevertheless, individual loading uncertainty remained substantial in the retained three-component solution. Of the 45 individual loading intervals, only two excluded zero. These corresponded to Pulses (95% bootstrap interval: 0.136 to 0.851) and Vegetables (0.002 to 0.785) within the Plant-rich component. Several other prominent loadings had wide intervals spanning zero (Figure S3), indicating that the contribution of individual food groups may vary across resampled datasets. Thus, the three-component solution showed comparatively greater overall reproducibility, but the precise allocation of individual food groups should be interpreted cautiously.

Within the retained three-component solution, the Plant-rich component explained 16.30% of the variance, the Animal-source foods component 15.18%, and the Energy-dense/discretionary component 14.76%.

The first component was labelled Plant-rich. Its strongest positive loadings were Pulses (0.790), Nuts/Seeds (0.670), Vegetables (0.661), Cereals/Grains (0.619), and Fruit (0.539), with a smaller positive loading for Fish/Seafood (0.341).

The second component was labelled Animal-source foods and was characterised principally by Red/processed meat (0.766), Poultry (0.765), Fish/Seafood (0.649), and Eggs (0.531). Dairy/Alternatives also loaded positively (0.380).

The third component, labelled Energy-dense/discretionary, was characterised by Fats/Oils (0.788), Discretionary foods (0.648), Soft/energy/sports drinks (0.551), Alcoholic beverages (0.480), Cereals/Grains (0.450), and Dairy/Alternatives (0.448).

**Figure 1:**
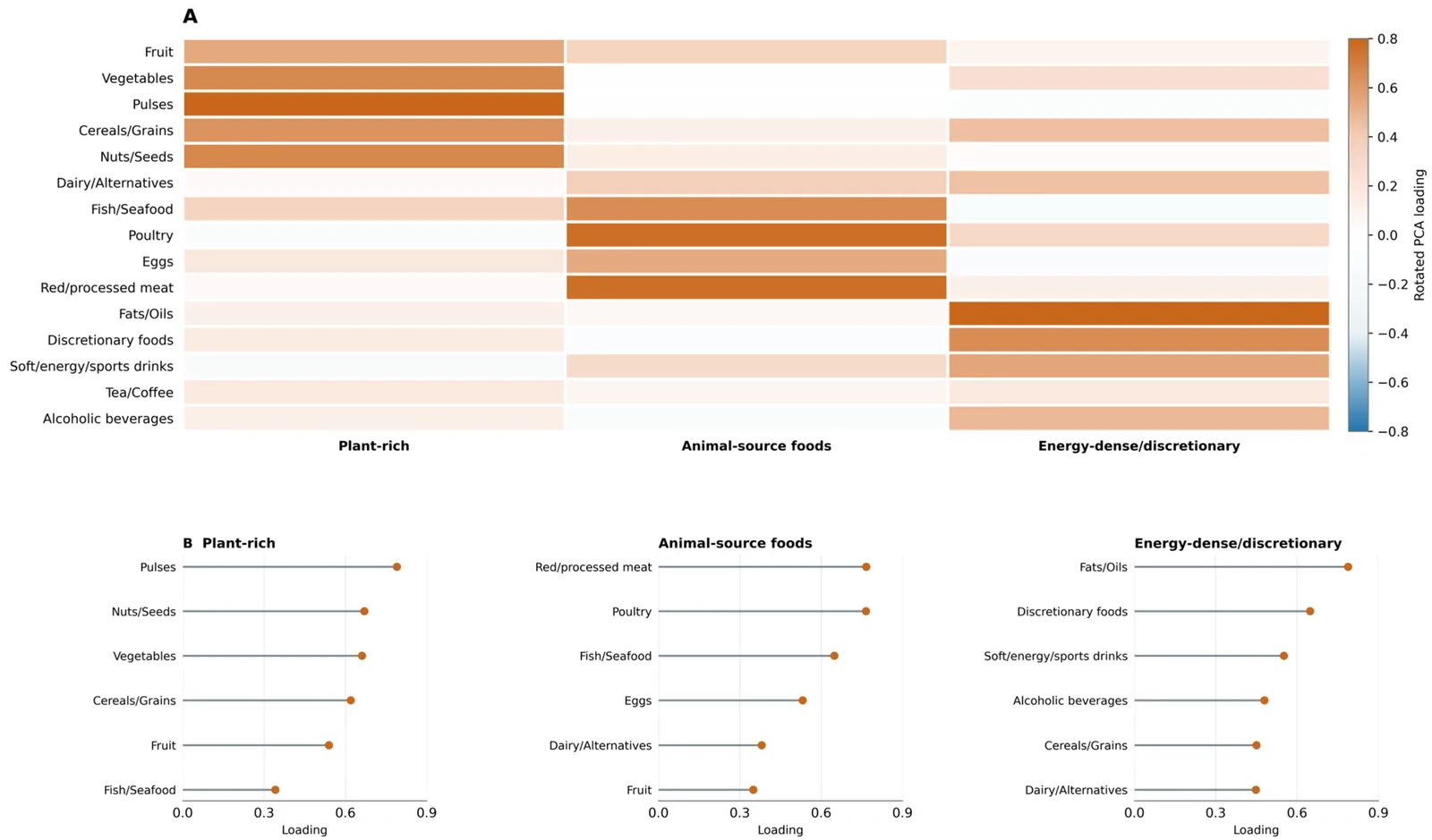
Rotated loading structure of the three dietary patterns identified by principal component analysis (PCA) of 15 analytical food groups. (**A**) Heatmap showing the signed food-group loadings across the three retained components. Warm and cool colours indicate positive and negative loadings, respectively, with colour intensity reflecting loading magnitude. (**B**) Dot plots highlighting the principal positive food-group loadings characterising each dietary pattern. Each dot represents the rotated loading coefficient of the corresponding food group. PCA was performed on standardised log1p-transformed daily food-group intakes, followed by orthogonal varimax rotation with Kaiser normalisation. Pattern labels were assigned descriptively according to their loading structure. Exact loading values, communalities, and variance explained are provided in Table 3.

The signed loading structure is visualised in Figure 1, while exact loadings, communalities, and variance explained are reported in Table 3. Of note, Tea/Coffee had a low communality (0.067), indicating that its variation was poorly captured by the retained three-component structure and should not be strongly attributed to any of the identified patterns.

### 3.4. Dietary-Pattern Scores According to Sex and Geographical Background

**Figure 2:**
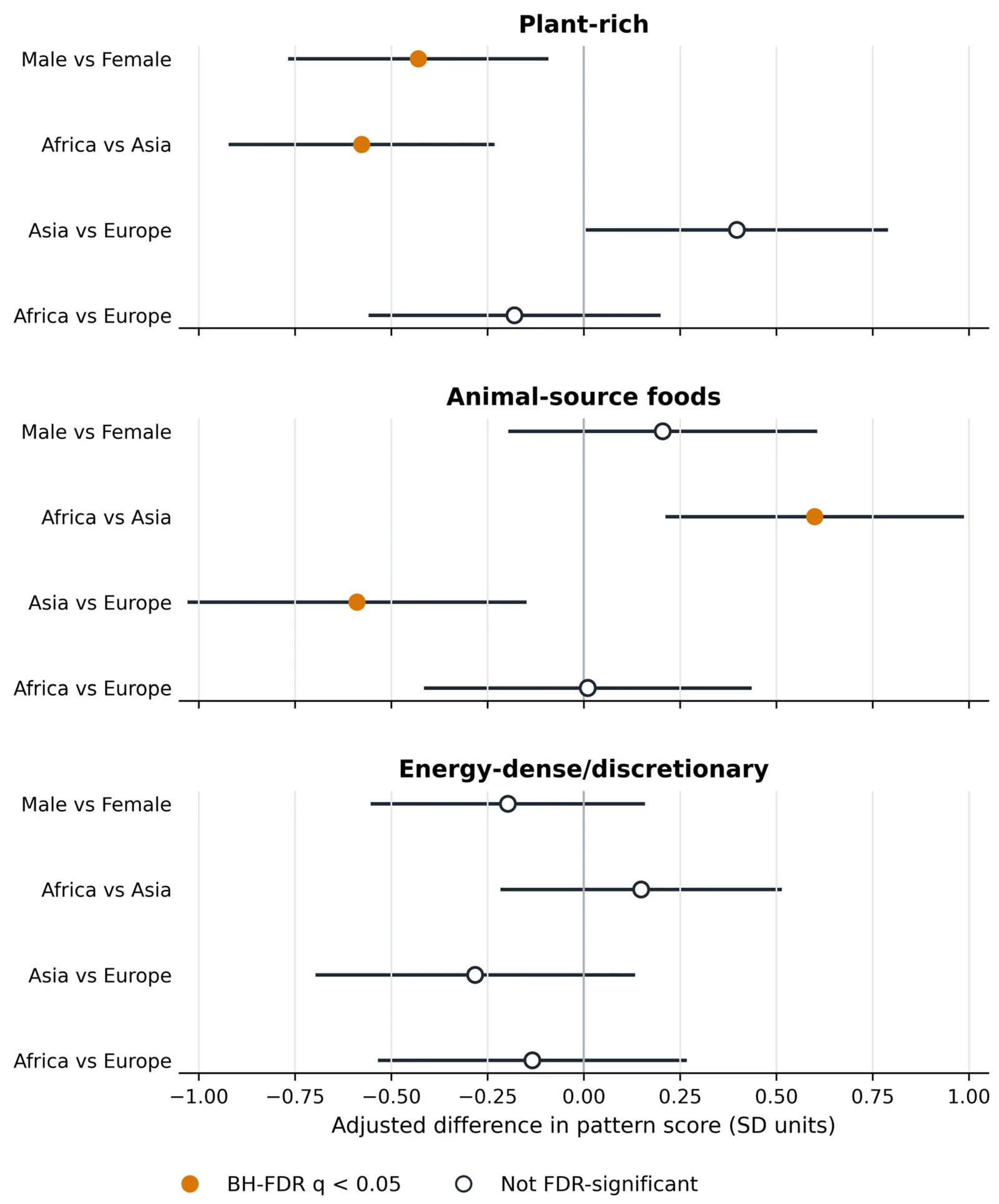
Adjusted differences in dietary-pattern scores according to sex and geographical background. Estimates are standardised differences (SD units) with 95% confidence intervals. Sex models were adjusted for age, geographical background and log-transformed total energy intake; geographical-background models were adjusted for sex, age and log-transformed total energy intake, with the Other category excluded from formal inference. Filled markers indicate comparisons surviving Benjamini–Hochberg false-discovery-rate correction (BH-FDR *q* < 0.05); open markers did not survive FDR correction. Positive estimates indicate higher scores in the first-named group relative to the second-named group.

Adjusted dietary-pattern comparisons are shown in Figure 2, and the complete model outputs are provided in Tables S4–S6.

After adjustment for age, geographical background, and log-transformed total energy intake, males had a lower Plant-rich pattern score than females (adjusted difference = −0.43 SD; 95% CI −0.76 to −0.10; *p* = 0.01; *q* = 0.04).

This effect warrants a compositional interpretation. The corresponding unadjusted sex effect was negligible (Hedges’ *g* = +0.01). Thus, the adjusted result indicates a lower relative Plant-rich dietary composition among males at comparable total energy intake, rather than demonstrating universally lower absolute consumption of plant foods.

No sex difference survived BH-FDR correction for the Animal-source foods pattern (+0.20 SD; 95% CI −0.19 to +0.60; *q* = 0.31) or the Energy-dense/discretionary pattern (−0.20 SD; 95% CI −0.55 to +0.15; *q* = 0.31).

Geographical background showed a global association with the Plant-rich pattern (*F* = 5.75; partial η^2^ = 0.101; *p* = 0.004; *q* = 0.006). In the FDR-adjusted pairwise analysis, participants in the Africa group had lower Plant-rich scores than participants in the Asia group (−0.58 SD; 95% CI −0.92 to −0.24; *p* = 0.001; *q* = 0.01). The Asia–Europe contrast did not survive FDR correction.

The Animal-source foods pattern also differed globally according to geographical background (*F* = 5.75; partial η^2^ = 0.100; *p* = 0.004; *q* = 0.006). Participants from Africa had higher adjusted scores than those from Asia (+0.60 SD; 95% CI +0.22 to +0.98; *p* = 0.002; *q* = 0.01), while participants from Asia had lower scores than those from Europe (−0.59 SD; 95% CI −1.02 to −0.15; *p* = 0.009; *q* = 0.03).

No robust global geographical-background difference was observed for the Energy-dense/discretionary pattern (*F* = 0.95; partial η^2^ = 0.018; *p* = 0.39; *q* = 0.39).

### 3.5. Food-Group and Core-Diet Differences

**Figure 3:**
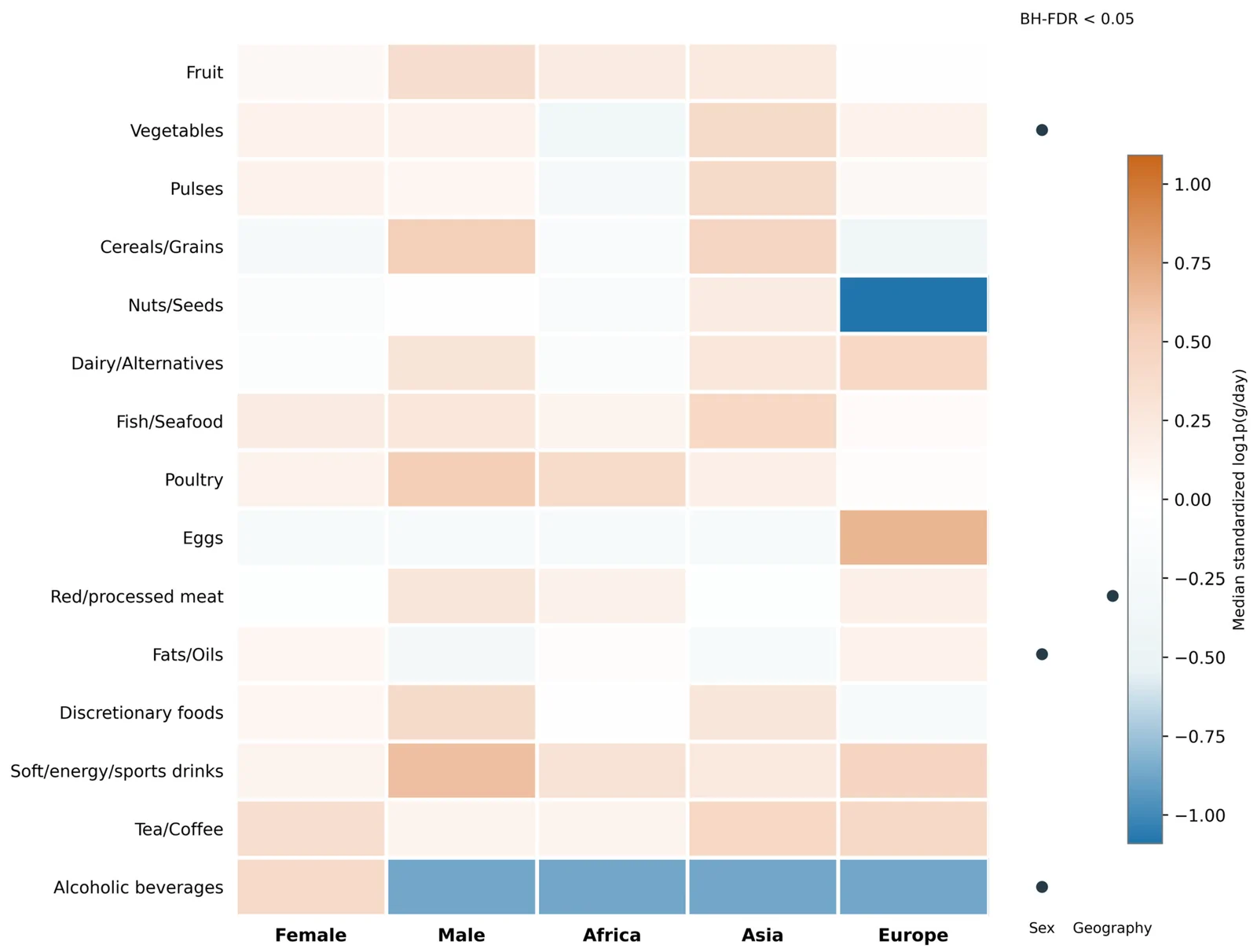
Food-group dietary landscape according to sex and geographical background. Cells show median standardised log1p-transformed intake for each food group within each stratum. Black dots in the annotation columns on the right indicate statistically significant differences after Benjamini–Hochberg false-discovery-rate correction (BH-FDR; *q* < 0.05). Dots in the Sex column represent significant adjusted sex differences, whereas dots in the Geography column indicate significant global geographical-background differences. The dots refer to the corresponding food group as a whole and do not represent cell-specific comparisons. Geographical-background summaries display Africa, Asia and Europe; the two participants classified as Other were excluded from formal geographical inference.

The food-group dietary landscape according to sex and geographical background is shown in Figure 3. Following covariate adjustment and BH-FDR correction, three individual food groups showed robust sex differences.

Relative to females, males had lower energy-adjusted standardised intake of Alcoholic beverages (−0.64 SD; 95% CI −1.05 to −0.23; *p* = 0.002; *q* = 0.02), Fats/Oils (−0.59 SD; 95% CI −0.97 to −0.20; *p* = 0.003; *q* = 0.02), and Vegetables (−0.59 SD; 95% CI −0.95 to −0.23; *p* = 0.002; *q* = 0.02).

Among the 15 individual food groups, Red/processed meat showed the clearest geographical-background signal after correction for multiple testing (F = 7.41; partial η^2^ = 0.126; *p* = 0.001; *q* = 0.02). Participants from Asia had lower adjusted Red/processed-meat intake than those from Europe (−0.72 SD; 95% CI −1.16 to −0.27; *p* = 0.002; *q* = 0.04) and lower intake than those from Africa, expressed equivalently by an Africa–Asia difference of +0.67 SD (95% CI +0.28 to +1.06; *p* = 0.0009; *q* = 0.04).

Complete adjusted food-group comparisons are provided in Tables S4–S6, with the contrasts surviving BH-FDR summarised in Figure S4.

Core-diet analyses further illustrated the importance of distinguishing absolute intake from energy-adjusted dietary composition. Males had higher total energy intake than females after adjustment for age and geographical background (+0.67 SD; 95% CI +0.28 to +1.06; *p* = 0.001; *q* = 0.005).

For total fat, the unadjusted sex effect suggested higher absolute intake in males (Hedges’ *g* = +0.41), whereas the energy-adjusted model showed a lower relative total-fat intake (−0.24 SD; 95% CI −0.39 to −0.09; *p* = 0.002; *q* = 0.005). These estimates address different questions and were therefore not interpreted as contradictory. Geographical background was also associated with energy-adjusted protein, total-fat, and fibre intake in the global models (BH-FDR *q* = 0.04 for each outcome). Given the whole-diet focus of the present study, these nutrient-level findings were considered secondary; complete global and pairwise estimates are provided in Tables S5 and S6.

## 4. Discussion

### 4.1. Principal Findings

This study identified three distinct and interpretable dietary dimensions—Plant-rich, Animal-source foods, and Energy-dense/discretionary—in a diverse cohort of young adults attending a UK university. Together, the patterns explained 46.25% of variation across the 15 analytical food groups.

The principal demographic signals were not uniformly distributed across the three patterns. Plant-rich scores differed according to sex and geographical background after multivariable and energy adjustment, while Animal-source-food scores differed according to geographical background. In contrast, the Energy-dense/discretionary pattern showed no robust differences by either sex or geographical background after correction for multiple comparisons.

This structure is important because it does not support a simple division of participants into universally “healthy” and “unhealthy” diets. PCA scores represent continuous, coexisting dimensions: the same participant can simultaneously express elements of more than one pattern [8,9]. Plant-oriented consumption, animal-source consumption, and discretionary/energy-dense consumption therefore appear to represent partly independent dimensions of dietary behaviour in this cohort.

From a nutritional perspective, the three patterns may capture potentially relevant differences in food selection and nutrient exposure. The Plant-rich pattern was characterised by the co-consumption of pulses, nuts/seeds, vegetables, cereal products, and fruit, which may contribute dietary fibre and a range of essential micronutrients. The Animal-source foods pattern reflected greater consumption of meat, poultry, fish, and eggs, which can contribute high-quality protein and nutrients such as vitamin B12, iron, and zinc. However, its nutritional implications depend on the specific foods consumed, their preparation, and the relative contribution of processed meat and other animal-source products. The Energy-dense/discretionary pattern combined fats/oils, Discretionary foods, and selected beverages, but its label should not be interpreted as a direct measure of dietary quality because the nutritional composition of these categories was heterogeneous.

Accordingly, none of the three patterns can be regarded as inherently beneficial or harmful on the basis of its PCA label alone. The present study did not establish clinical outcomes, micronutrient adequacy, or long-term disease risk associated with these patterns. Their principal value lies in identifying empirically derived dietary dimensions that can be evaluated more specifically in future nutritional and epidemiological investigations.

### 4.2. Comparison with Previous Dietary-Pattern Studies in University Students

The present results can be interpreted alongside previous UK university research, particularly the study by Sprake et al. [10]. In that multicentre sample of 1448 students, four PCA-derived patterns were described as vegetarian, snacking, health-conscious, and convenience/red meat/alcohol. Female students showed greater adherence to the vegetarian pattern, while males showed greater adherence to the convenience/red meat/alcohol pattern [10].

The current Plant-rich pattern shares conceptual features with both the vegetarian and health-conscious dimensions reported by Sprake et al., particularly its positive loadings for pulses, nuts/seeds, vegetables, cereals/grains, and fruit. Likewise, our Energy-dense/discretionary pattern incorporates several features conceptually related to convenience/snacking-type behaviour, including Discretionary foods, fats/oils, soft/energy/sports drinks, and Alcoholic beverages.

However, these patterns should not be treated as directly interchangeable. Empirical dietary patterns depend strongly on the structure of the questionnaire, number and composition of food groups, transformation and scaling decisions, sample characteristics, and the component-retention strategy [8,9,10]. The present analysis also identified a distinct Animal-source foods dimension dominated by red/processed meat, poultry, fish/seafood, and eggs. This suggests that, in the DMU cohort, animal-source food consumption formed a coherent dietary dimension not fully subsumed into a general convenience or energy-dense pattern.

Tanton et al. similarly demonstrated marked heterogeneity among British university students, although using a person-centred cluster approach rather than PCA [4]. Only a minority of participants fell within their most favourable eating-behaviour cluster, while substantial proportions displayed mixed or riskier patterns [4]. Cluster and component analyses should not be viewed as competing methods: clustering partitions participants into groups, whereas PCA permits each participant to express multiple dietary dimensions simultaneously [8,9]. For the present research question, the continuous-score approach is particularly useful because dietary behaviours in a geographically diverse cohort are unlikely to fall naturally into mutually exclusive categories.

More recent university studies further support the need to consider diet as multidimensional. Multivariate segmentation has identified distinct profiles of poor diet quality across international university populations [6], while contextual research shows that dietary identity, food setting, and decision-making motives can influence consumption in category-specific ways [7]. Collectively, these findings reinforce the premise that a single summary description such as “the student diet” is unlikely to capture the heterogeneity of university food behaviour.

### 4.3. Sex Differences and the Importance of Energy Adjustment

One of the strongest findings was the lower adjusted Plant-rich score observed in males. This is broadly compatible with previous UK evidence showing greater female adherence to vegetarian or more health-conscious dietary patterns [10]. However, the present result requires an important qualification: the unadjusted Plant-rich sex effect was essentially null, whereas the difference became evident after adjustment for total energy intake.

Energy adjustment is used in nutritional epidemiology to distinguish differences in diet composition from differences attributable to overall quantity consumed [30]. This distinction is particularly relevant because males in the present study had substantially higher total energy intake. A participant consuming more total food may consume more grams of both plant and animal foods without having a greater *relative* dietary emphasis on those foods.

The adjusted Plant-rich coefficient should therefore be interpreted as indicating that, at comparable total energy intake, males had a lower relative expression of the Plant-rich dietary pattern. It should not be translated into the simpler claim that males consumed less plant food in absolute terms.

The total-fat results provide a second illustration. Absolute intake was higher in males before energy adjustment, but the adjusted coefficient was negative. These findings are not contradictory: the former reflects quantity, whereas the latter reflects dietary composition conditional on energy intake.

This distinction is important because failure to separate absolute from energy-adjusted estimates can generate apparently inconsistent conclusions and may obscure the dietary behaviour actually represented by the statistical model [30].

Adjusted sex differences were also identified for Alcoholic beverages, Fats/Oils, and Vegetables. These food-specific findings should nevertheless be interpreted as secondary to the broader dietary-pattern results. The sample was relatively small, and the objective was not to construct a catalogue of sex differences across every food group. The use of predefined outcome families and BH-FDR correction was intended specifically to reduce such overinterpretation.

### 4.4. Geographical Background and Dietary Heterogeneity

Both the Plant-rich and Animal-source-food patterns differed according to geographical background. Participants classified within the Asia group had higher Plant-rich scores than those in the Africa group, while Animal-source-food scores were lower in the Asia group than in both the Africa and Europe groups. Red/processed meat was the individual food group showing the clearest corresponding geographical-background effect.

These findings may reflect differences in dietary practices and food environments within the geographically diverse university population. Cultural food traditions, religious dietary practices, family eating habits, and access to familiar ingredients could plausibly influence the consumption of plant-based and animal-source foods. Gender-related influences, living arrangements, financial resources, and migration or acculturation experiences may also contribute to dietary variation, as suggested by previous research on university food choice [2,6,7].

However, the study recorded broad geographical background rather than detailed sociocultural determinants. These potential explanations should therefore be regarded as contextual hypotheses, not established mechanisms underlying the observed associations. Future studies incorporating more specific measures of dietary practices and social circumstances could help explain the differences identified here.

### 4.5. Nutritional Context and UK Dietary Recommendations

The present study was designed as a whole-diet pattern analysis rather than a formal dietary-adequacy study. Nevertheless, the descriptive results provide useful context when considered against UK recommendations.

Median fibre intake was 25.4 g/day, below the current SACN population recommendation of approximately 30 g/day for individuals aged 16 years and over [12]. This comparison should not be interpreted as demonstrating individual fibre deficiency. Dietary reference values are intended primarily to guide and evaluate population intake, and habitual intake estimated by FFQ contains both random and systematic measurement error.

Micronutrient intake is another important consideration in this age group, particularly for nutrients such as vitamin D, vitamin B12, iron, and zinc. These nutrients contribute to physiological functions including bone health, haematopoiesis, and normal metabolic and immune processes. Different food-group combinations may provide distinct micronutrient profiles, with animal-source foods, plant foods, fortified products, and dietary supplements contributing to nutrient intake in different ways. Previous investigations within this cohort have addressed selected nutrients, including vitamin D and iron [23,25]. However, the present analysis did not undertake a comprehensive assessment of micronutrient adequacy, and the identified PCA patterns should not be interpreted as evidence of adequate or inadequate vitamin or mineral status.

Similarly, the additional descriptive information on saturated, monounsaturated, and polyunsaturated fatty acids and total sugars provides context for dietary composition without establishing individual dietary risk. In particular, total sugars include naturally occurring and free sugars and should not be compared directly with recommendations specifically concerning free-sugar intake.

The food-group structure also showed substantial consumption of fruit, vegetables, cereals/grains, dairy/alternatives, and pulses, which are relevant to the overall balance represented by the Eatwell Guide [11]. However, the analytical food groups used here do not map one-to-one onto Eatwell Guide portions. For example, the Fruit group includes selected juice-derived entries, and mixed foods were handled through predefined mapping rules. Consequently, the present data should not be used to claim formal adherence to the “5 A Day” recommendation without a dedicated compliance analysis.

The same caution applies to Soft/energy/sports drinks. This analytical group was intentionally broad for the present whole-diet analysis and included beverages with differing sugar profiles. It should therefore not be interpreted as synonymous with sugar-sweetened beverages, and no sugar-specific conclusions are drawn from this food-group classification. Accordingly, the present analysis focused on food-group structure and whole-diet patterns rather than attempting simultaneous inference across the complete nutrient dataset. This restriction maintained alignment between the statistical analysis and the predefined whole-diet research question while limiting unnecessary multiplicity.

### 4.6. Historical and Contemporary UK Context

The timing of data collection requires explicit consideration. Participants were assessed during early 2016, making the cohort particularly suitable for contextualisation against the NDNS Years 7 and 8 rolling programme, covering 2014/15 and 2015/16 [13]. This survey therefore provides the most temporally aligned nationally representative UK context for the original DMU data.

The more recent NDNS 2019–2023 report provides contemporary context but should not be used for direct temporal comparison with the present cohort [14]. From 2019, NDNS changed its dietary-assessment methodology from a paper diary to the online *Intake24 system*, producing step changes in estimates for some foods and nutrients; the official report consequently cautions against direct comparison of 2019–2023 dietary estimates with those from earlier survey years. The contemporary NDNS data are therefore used here for contextual orientation rather than formal trend assessment.

The DMU cohort should therefore be understood as a historical cross-sectional university cohort. The results describe dietary behaviour within this specific population during the mid-2010s; they do not estimate current prevalence of particular dietary patterns among UK university students in 2026.

This temporal framing is also valuable scientifically. University food environments, prices, product availability, delivery services, plant-based food markets, and public-health messaging have changed appreciably since the original data were collected. The present results can therefore provide a well-characterised historical reference against which appropriately designed future student cohorts could be compared.

### 4.7. Methodological Strengths

Several features strengthen the present analysis.

First, participant-level dietary information was reconstructed from the original source database, preserving traceability from individual food records through normalised dietary categories and final analytical food groups. Raw and derived variables were retained separately, providing a transparent and reproducible audit trail.

Second, dietary units were independently verified before conversion, and weekly food-intake values were converted to daily intake only for variables documented as weekly measures. This source-based reconstruction reduced reliance on previously processed datasets and ensured consistent units across the final analytical dataset.

Third, food-group mapping was explicitly audited. Potentially ambiguous foods were reviewed at *FoodName* level rather than assigned automatically according to a broad historical category. This was particularly important for mixed products, poultry, fried potato products, grain products, beverages, and food groups used in the PCA.

Fourth, the PCA was not selected through a single mechanical criterion. The transformed and untransformed matrices were compared; KMO, Bartlett’s test, correlation structure, parallel analysis, scree/eigenvalue behaviour, loading interpretability, and bootstrap stability were considered together. This strategy reduces the likelihood of retaining components solely because they meet an arbitrary eigenvalue threshold [26,27,28].

Fifth, the statistical analysis emphasised effect sizes and 95% CIs alongside *p*-values, and BH-FDR correction was conducted within predefined outcome families [31,32]. This was particularly important given the number of food-group comparisons.

Finally, the cohort included measured anthropometric and body-composition information rather than relying exclusively on self-reported weight and height. These data permit detailed nutritional phenotyping while allowing the present paper to maintain a clear whole-diet focus.

### 4.8. Limitations

Several limitations should be considered when interpreting these findings. The study involved a modest sample of 111 volunteers from a single university, with greater participation by females than males. Recruitment through multiple overlapping communication channels precluded calculation of a formal response rate, and complete historical screening and exclusion counts were not available. As participation may have been associated with characteristics such as health awareness or interest in nutrition, some selection bias cannot be excluded. These considerations primarily affect the representativeness and generalisability of the cohort rather than the validity of the descriptive findings within the analysed sample.

Diet was assessed using an FFQ and is therefore susceptible to recall error, portion-size estimation error, food-composition error, and systematic misreporting [19,21]. Although the questionnaire was based on the established EPIC-Norfolk FFQ, the adaptations introduced to improve food coverage in this geographically diverse university population were not independently validated against a reference dietary-assessment method in the present cohort. Accordingly, the instrument should be regarded as an adapted FFQ rather than as a newly validated population-specific instrument. Although energy-intake plausibility was examined during quality control, sufficiently complete physical-activity information was not available to assign defensible individual PAL values, and formal individual Goldberg classification was therefore not undertaken [33,34].

Subsequent methodological research at De Montfort University further demonstrated the feasibility of adapting EPIC-Norfolk-derived questionnaires to improve the dietary coverage of multiethnic populations in Leicester, including additional South Asian foods and customised food-code processing [36]. However, that investigation employed a different questionnaire and study population and does not establish the validity of the expanded FFQ used in the present cohort.

Body-composition variables were estimated using BIA rather than a reference method such as dual-energy X-ray absorptiometry. Although premeasurement conditions were standardised by requiring at least 8 h without food, alcohol, coffee, smoking, or physical exercise, with water permitted, BIA estimates may still be influenced by individual hydration status and other physiological factors [17].

PCA is an exploratory, sample-dependent method, and the resulting patterns may vary according to food-group definitions, analytical transformations, and population characteristics [8,9,28]. Although parallel analysis and bootstrap assessment supported retention of three components, the wide bootstrap intervals for several individual loadings indicate uncertainty in their precise food-group composition. Independent replication would therefore be valuable to assess the generalisability and stability of these dietary dimensions.

The retained patterns also did not capture all food groups equally. In particular, Tea/Coffee had a communality of only 0.067, indicating that its variation was largely outside the three-dimensional dietary structure identified here.

The geographical-background variable is necessarily coarse. Africa, Asia, and Europe comprise heterogeneous regions, and geographical background should not be treated as equivalent to ethnicity or culture. The Other category contained only two participants and could not be included in formal geographical inference.

This study is also cross-sectional. No temporal or causal interpretation can be made from associations between participant characteristics and dietary-pattern scores.

Finally, the data were collected in early 2016. Although this provides a useful historical cohort and enables comparison with temporally aligned NDNS data [13], it limits direct inference about the present-day dietary behaviour of UK students.

### 4.9. Implications and Future Research

Despite these limitations, the findings emphasise the heterogeneity of dietary behaviour within a single university population. University nutrition strategies should therefore avoid assuming that students constitute a homogeneous dietary group.

The coexistence of Plant-rich, Animal-source-food, and Energy-dense/discretionary dimensions suggests that intervention design may benefit from focusing on specific dietary behaviours and food environments rather than attempting to assign individuals to simplistic “healthy” or “unhealthy” categories.

The broader public-health relevance of dietary behaviour in emerging adulthood also extends to disordered eating and the longer-term development of non-communicable diseases. University students may experience changes in food access, eating routines, body-image concerns, and social pressures that could influence both dietary quality and eating behaviour [37]. Furthermore, sustained dietary exposures characterised by inadequate consumption of protective food groups or excessive intake of certain dietary components may contribute to long-term cardiometabolic and other non-communicable disease risks [38].

Nevertheless, the present study did not assess eating-disorder symptoms, psychiatric diagnoses, prospective disease outcomes, or the longitudinal persistence of the identified patterns. No relationship between the present PCA scores and eating disorders or chronic disease risk can therefore be established. Future longitudinal investigations incorporating validated assessments of disordered eating, more detailed dietary-quality indicators, and clinical outcomes would be necessary to explore these potential relationships.

From a research perspective, future multicentre studies should combine detailed food-group data with longitudinal assessment, socioeconomic circumstances, food security, living arrangements, cooking skills, institutional food-environment measures, and more detailed cultural or migration variables. Replication of the present PCA structure would also be valuable.

## 5. Conclusions

Three empirically derived dietary patterns—Plant-rich, Animal-source foods, and Energy-dense/discretionary—were identified among 111 young adults attending a diverse UK university and collectively explained 46.25% of food-group variation.

After multivariable and total-energy adjustment, Plant-rich pattern scores differed according to sex and geographical background, while Animal-source-food pattern scores differed according to geographical background. No robust sex or geographical-background differences were identified for the Energy-dense/discretionary pattern following correction for multiple comparisons.

These findings indicate that food behaviour within this cohort was multidimensional and cannot be adequately represented by a single “student diet”. The results also demonstrate the importance of distinguishing absolute dietary intake from energy-adjusted dietary composition and of interpreting broad geographical-background differences cautiously.

Because this was a modest, single-centre, historical cross-sectional cohort, the findings should be regarded as a detailed characterisation of the study population rather than evidence of causality or current national prevalence. Replication in larger, contemporary, multicentre cohorts will be necessary to determine the stability and broader relevance of the dietary patterns identified.

Overall, this study provides a detailed historical reference for understanding dietary heterogeneity in geographically diverse university populations and highlights the value of multidimensional food-group assessment for informing future nutritional research and more appropriately targeted university health-promotion strategies.

## Figures and Tables

**Table 1:** Characteristics of the study population overall and by sex and geographical background (*N* = 111).

Characteristic	Unit	Overall (*N* = 111)	Female (*n* = 78)	Male (*n* = 33)	Africa (*n* = 41)	Asia (*n* = 41)	Europe (*n* = 27)	Other (*n* = 2)
**Female participants**	*n*	78	78	0	31	28	18	1
**Male participants**	*n*	33	0	33	10	13	9	1
**Age**	years	20.4 (1.2)	20.5 (1.1)	20.2 (1.2)	20.4 (0.9)	20.8 (1.3)	20.0 (1.2)	20.5 (0.7)
**Height**	cm	167.2 (9.3)	163.3 (7.0)	176.3 (7.5)	168.2 (8.3)	164.0 (8.2)	170.7 (11.0)	164.8 (4.5)
**Weight**	kg	66.4 (13.5)	64.4 (13.6)	71.3 (12.2)	66.2 (10.3)	62.7 (15.6)	72.2 (13.2)	68.3 (0.6)
**BMI**	kg/m^2^	23.7 (4.1)	24.0 (4.3)	22.9 (3.6)	23.4 (2.8)	23.1 (4.4)	24.9 (5.1)	25.2 (1.2)
**Body fat**	%	25.7 (10.4)	30.0 (9.0)	15.7 (5.6)	27.1 (10.2)	24.0 (10.6)	26.3 (10.8)	25.8 (9.8)
**Body water**	%	53.0 (6.7)	50.4 (5.7)	59.1 (4.5)	52.4 (6.3)	54.3 (7.1)	51.7 (6.5)	53.6 (8.3)
**Muscle mass**	kg	46.4 (8.8)	42.1 (5.1)	56.6 (7.3)	45.3 (7.1)	45.1 (9.9)	49.9 (9.1)	48.0 (6.0)
**Bone mass**	kg	2.5 (0.4)	2.2 (0.3)	3.0 (0.4)	2.4 (0.4)	2.4 (0.5)	2.6 (0.4)	2.5 (0.4)
**Visceral fat index**	index	2.7 (2.0)	2.7 (2.1)	2.6 (1.7)	2.4 (1.5)	2.6 (2.0)	3.1 (2.5)	5.0 (1.4)
**BMR**	kcal/day	1514.2 (256.0)	1400.1 (162.4)	1783.9 (234.9)	1484.9 (195.5)	1469.6 (294.9)	1623.3 (258.8)	1554.5 (137.9)
**Systolic BP**	mmHg	119.0 (13.7)	116.9 (14.3)	124.0 (10.7)	116.2 (13.5)	118.0 (13.7)	123.6 (10.6)	137.0 (35.4)
**Diastolic BP**	mmHg	69.7 (8.6)	70.6 (8.5)	67.7 (8.5)	69.6 (8.9)	69.9 (8.5)	70.3 (8.2)	61.5 (12.0)
**Pulse**	bpm	75.0 (11.4)	76.7 (11.2)	70.9 (10.9)	74.6 (10.5)	77.3 (13.6)	72.7 (8.5)	67.0 (7.1)

Values are mean (SD) for continuous characteristics and participant counts (n) for sex distribution. The female and male count rows provide the sex-by-geographical-background cross-tabulation. Cohort sex: 78 females (70.3%) and 33 males (29.7%). Geographical background: Africa, 41 (36.9%); Asia, 41 (36.9%); Europe, 27 (24.3%); Other, 2 (1.8%). Overall BMI categories: underweight, 10 (9.0%); normal weight, 64 (57.7%); overweight, 28 (25.2%); obesity, 9 (8.1%). BMI, body mass index; BMR, basal metabolic rate; BP, blood pressure. The Other group (*n* = 2) was retained in descriptive summaries but excluded from geographical-background inference.

**Table 2:** Overall dietary intake and food-group profile of the study population (*N* = 111).

Domain	Variable	Unit	Mean (SD)	Median [IQR]
Core diet	Energy intake	kcal/day	2524.1 (1256.1)	2245.4 [1664.1, 3114.5]
	Protein	g/day	118.3 (62.3)	104.2 [76.1, 140.0]
	Total fat	g/day	86.9 (45.4)	77.7 [54.6, 107.0]
	Saturated fatty acids (SFA)	g/day	31.1 (16.9)	26.9 [19.8, 36.1]
	Monounsaturated fatty acids (MUFA)	g/day	32.4 (16.2)	28.2 [21.6, 38.6]
	Polyunsaturated fatty acids (PUFA)	g/day	13.4 (7.5)	11.2 [8.3, 16.6]
	Carbohydrate	g/day	315.3 (166.7)	268.2 [195.4, 390.6]
	Total sugars	g/day	144.0 (91.7)	121.7 [83.4, 160.4]
	Fibre	g/day	28.6 (15.5)	25.4 [16.8, 37.0]
PCA food group	Alcoholic beverages	g/day	2.1 (3.1)	0.0 [0.0, 4.0]
	Cereals/Grains	g/day	335.1 (183.1)	291.9 [207.3, 436.7]
	Dairy/Alternatives	g/day	305.4 (276.3)	216.3 [123.6, 407.8]
	Discretionary foods	g/day	123.8 (99.5)	101.7 [62.5, 148.0]
	Eggs	g/day	16.9 (20.5)	7.1 [7.1, 21.4]
	Fats/Oils	g/day	5.5 (7.5)	3.4 [1.3, 7.4]
	Fish/Seafood	g/day	59.0 (66.9)	37.1 [10.7, 84.9]
	Fruit	g/day	272.9 (200.8)	239.3 [135.7, 346.6]
	Nuts/Seeds	g/day	15.0 (32.9)	3.4 [0.0, 12.7]
	Poultry	g/day	97.8 (82.3)	79.3 [41.8, 129.3]
	Pulses	g/day	61.6 (68.6)	39.4 [22.4, 72.1]
	Red/processed meat	g/day	115.9 (90.9)	87.7 [60.9, 164.3]
	Soft/energy/sports drinks	g/day	439.3 (558.1)	225.3 [70.7, 667.9]
	Tea/Coffee	g/day	236.4 (314.9)	111.4 [35.6, 286.5]
	Vegetables	g/day	339.3 (271.3)	277.4 [142.0, 449.2]

Values are presented as arithmetic mean (SD) and median [interquartile range (Q1, Q3)]. The four additional nutrient variables were recovered from the final validated nutritional dataset and were already expressed in g/day; no second weekly-to-daily conversion was applied. Food-group values follow the original validated PCA food-group mapping.

**Table 3:** PCA loadings, communalities and variance explained for the final three-pattern solution.

Food Group	Plant-Rich	Animal-Source Foods	Energy-Dense/Discretionary	Communality
**Alcoholic beverages**	0.124	−0.089	**0.480**	0.254
**Cereals/Grains**	**0.619**	0.115	**0.450**	0.600
**Dairy/Alternatives**	0.049	0.380	**0.448**	0.348
**Discretionary foods**	0.161	−0.064	**0.648**	0.450
**Eggs**	0.187	**0.531**	−0.101	0.328
**Fats/Oils**	0.114	0.055	**0.788**	0.638
**Fish/Seafood**	0.341	**0.649**	−0.114	0.550
**Fruit**	**0.539**	0.349	0.090	0.421
**Nuts/Seeds**	**0.670**	0.136	0.029	0.468
**Poultry**	−0.084	**0.765**	0.309	0.687
**Pulses**	**0.790**	0.004	−0.050	0.626
**Red/processed meat**	0.045	**0.766**	0.113	0.602
**Soft/energy/sports drinks**	−0.109	0.289	**0.551**	0.399
**Tea/Coffee**	0.178	0.079	0.171	0.067
**Vegetables**	**0.661**	−0.016	0.251	0.500
**Variance explained (%)**	16.30	15.18	14.76	-
**Cumulative variance (%)**	16.30	31.48	46.25	-

The three retained patterns explained 46.25% of total variance. Loadings with absolute values ≥ 0.40 are bolded in red. Communality represents the proportion of each food-group variance accounted for by the retained components.

## Data Availability

The derived, non-identifiable analytical outputs, food-group mapping information, and R code supporting the findings of this study are publicly available in Figshare at https://doi.org/10.6084/m9.figshare.33610678. The complete participant-level dataset is not publicly available because it contains human-participant information subject to ethical, institutional, and data-protection requirements. Requests for additional de-identified participant-level data may be considered by the corresponding author in accordance with the applicable ethical and institutional conditions.

## Supplementary Materials

The following supporting information can be downloaded at: https://www.mdpi.com/article/10.3390/nu18213503/s1, Figure S1: Reproducible reduction of participant-level dietary data used for dietary-pattern analysis; Table S1: Mapping of normalised dietary source categories to analytical food groups and PCA eligibility; Table S2: Two-component principal component analysis: rotated food-group loadings, communalities, and variance explained; Table S3: PCA suitability, component-retention diagnostics and variance explained; Table S4: Adjusted sex comparisons across cohort characteristics, core diet, food groups and dietary-pattern scores; Table S5: Adjusted global tests according to geographical background; Figure S2: Observed eigenvalues and the 95th-percentile reference from parallel analysis for the standardised log1p(g/day) food-group matrix; Figure S3: Bootstrap stability of food-group loadings for the retained three-pattern solution; Table S6: Adjusted pairwise geographical-background comparisons; Figure S4: Food-group contrasts that survived BH-FDR correction.
