# Supplementary material for: Dietary Patterns, Food-Group Profiles, and Nutritional Characteristics in a Diverse Cohort of Young Adults Attending a UK University (2016)
Source: Nutrients. 2026 Oct 25;18(21):3503. doi: 10.3390/nu18213503 (PMC13765290; doi:10.3390/nu18213503)
Supplement: Supplementary file 1 [file nutrients-18-03503-s001.zip › Supplementary Figures art_revised.pdf]

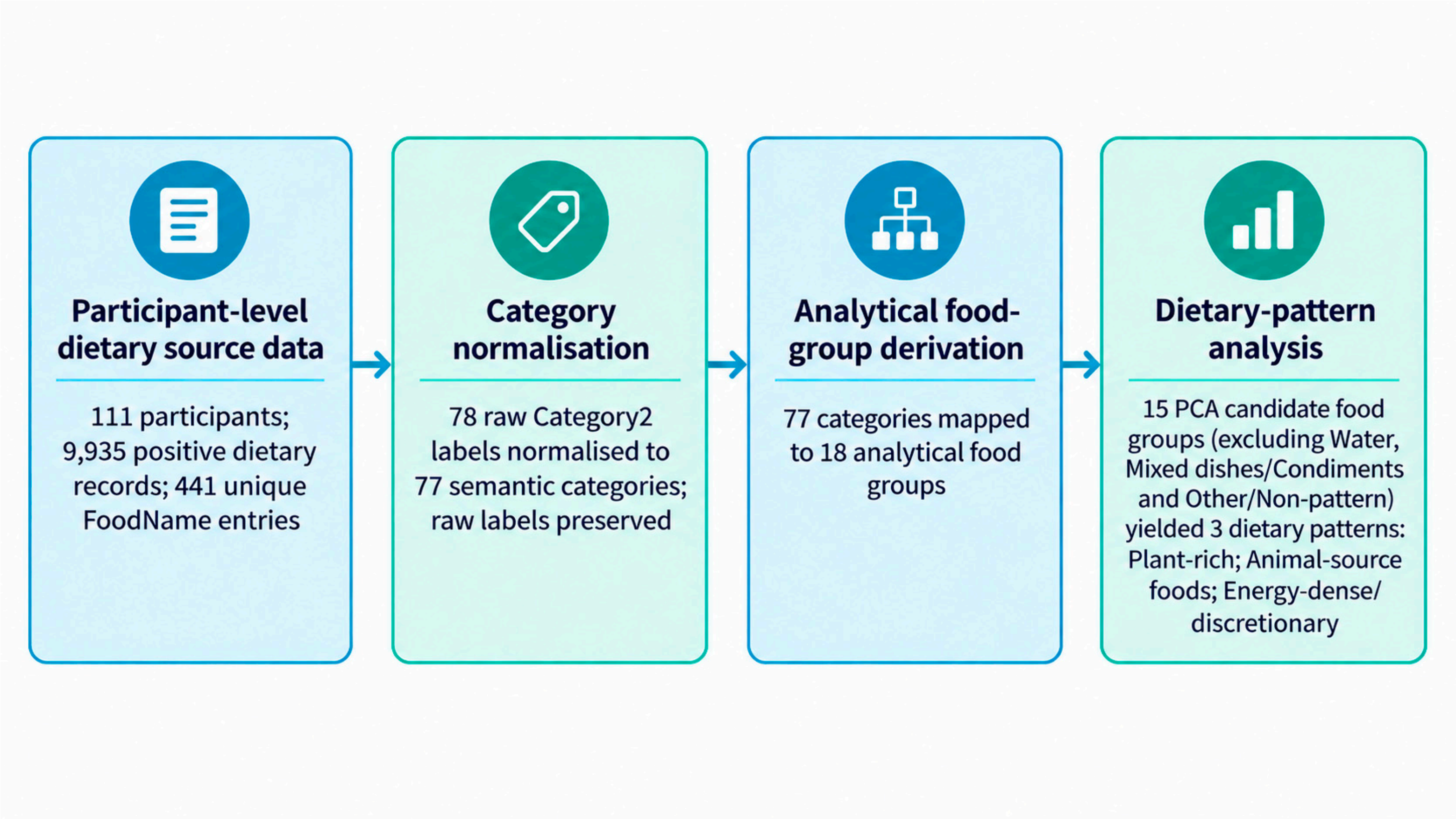

Figure S1. Reproducible reduction of participant-level dietary data used for dietary-pattern analysis. The original Access database contained 9,935 positive dietary records from 111 participants and 441 unique FoodName entries. Seventy-eight raw Category2 labels were normalised to 77 semantic categories while retaining the original raw labels. These categories were mapped to 18 analytical food groups; 15 food groups were retained as PCA candidates after excluding Water, Mixed dishes/Condiments, and Other/Non-pattern, yielding three final dietary patterns. Source-level provenance and the complete audit trail were preserved throughout; weekly gram values were converted to g/day only after independent unit validation.

.

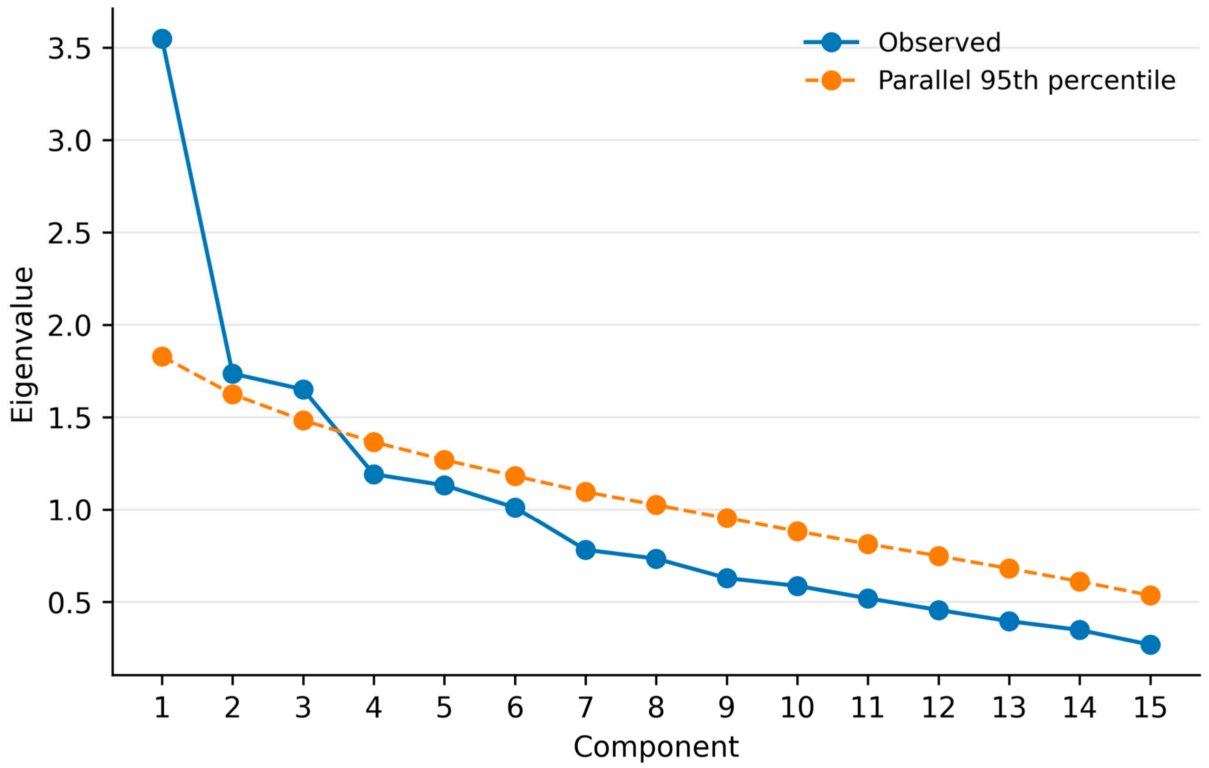

Figure S2. Observed eigenvalues and the 95th-percentile reference from parallel analysis for the standardized log1p(g/day) food-group matrix. The first three observed eigenvalues exceeded the corresponding parallel-analysis threshold.

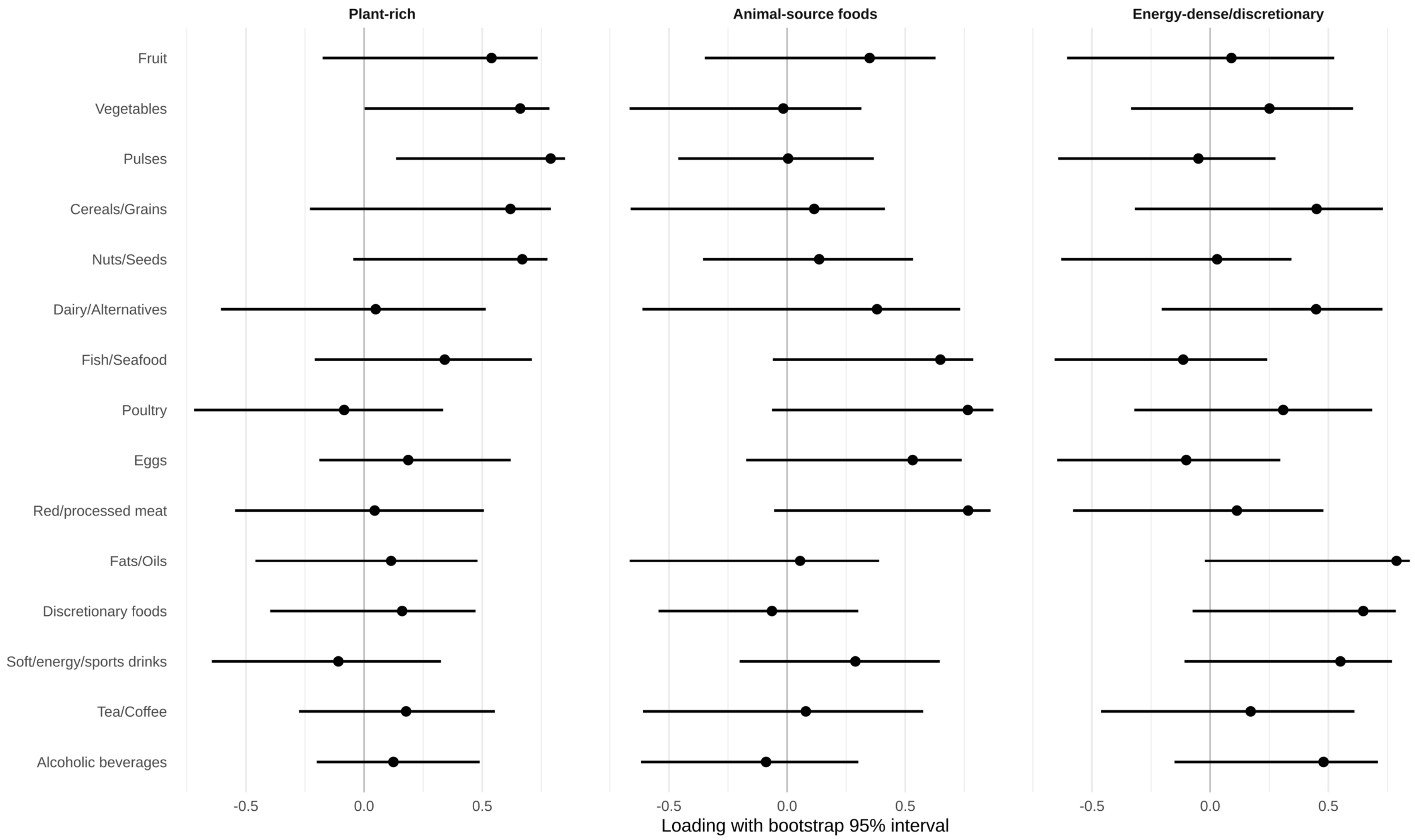

Figure S3. Bootstrap stability of food-group loadings for the retained three-pattern solution. Points show the final-solution loading and horizontal intervals show bootstrap 95% intervals. The plot is provided as a robustness diagnostic for pattern interpretation.

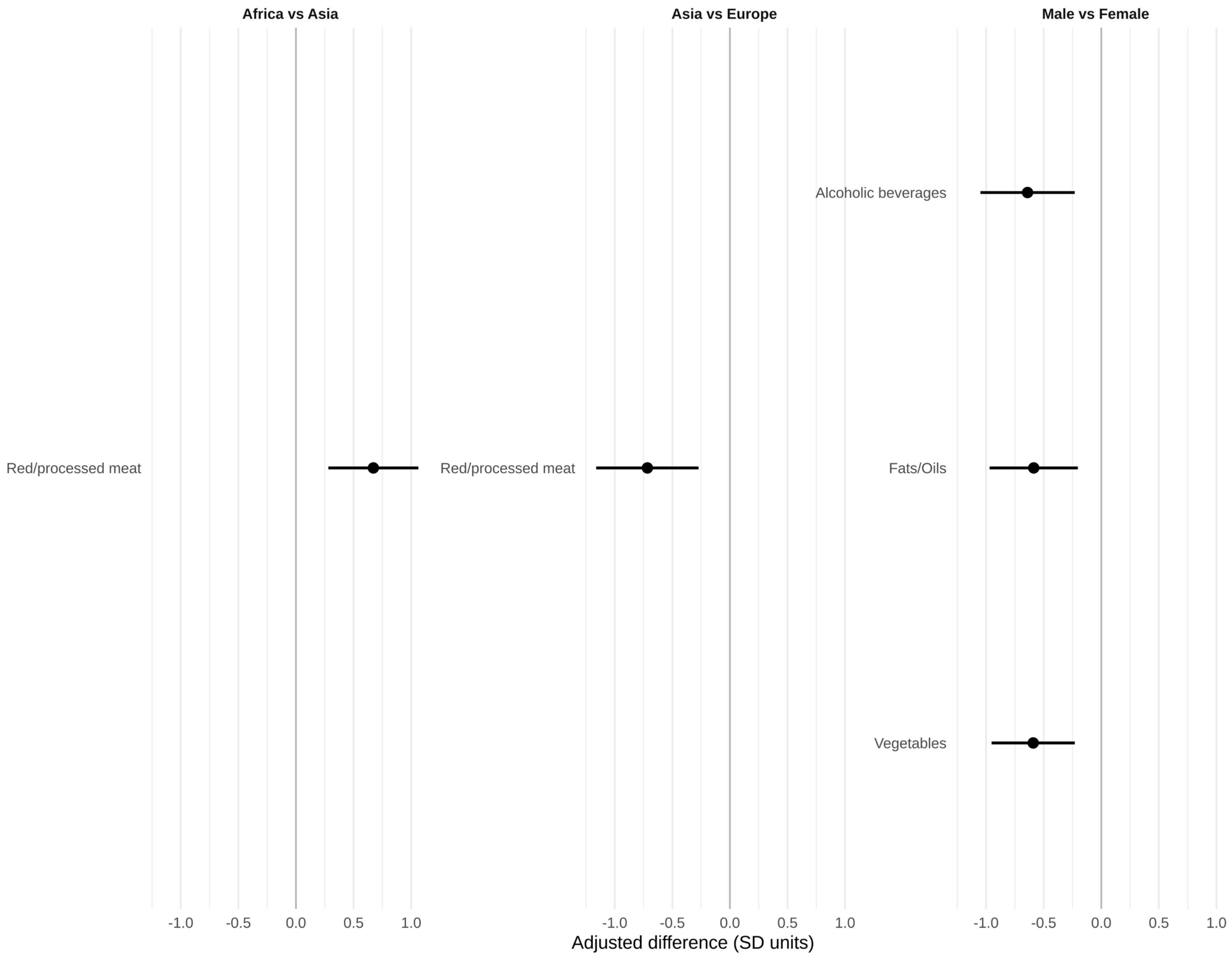

Figure S4. Food-group contrasts that survived BH-FDR correction. Points show adjusted standardized differences with 95% confidence intervals. Sex models were adjusted for age, geographical background and log-transformed total energy intake; geographical-background models were adjusted for sex, age and log-transformed total energy intake.
