# Supplementary material for: Dietary Patterns, Food-Group Profiles, and Nutritional Characteristics in a Diverse Cohort of Young Adults Attending a UK University (2016)
Source: Nutrients. 2026 Oct 25;18(21):3503. doi: 10.3390/nu18213503 (PMC13765290; doi:10.3390/nu18213503)
Supplement: Supplementary file 1 [file nutrients-18-03503-s001.zip › Supplementary Tables art_revised.pdf]

Table S1. Mapping of normalised dietary source categories to analytical food groups and PCA eligibility.

Normalised source category	Unique *FoodName* entries, *n*	Final analytical food group(s)	*FoodName*-level split	PCA eligibility
Alcoholic Drinks	1	Alcoholic beverages	No	Yes
Bacon	1	Red/processed meat	No	Yes
Beans ǀ Peas ǀ Lentils	19	Pulses	No	Yes
Beef	5	Red/processed meat	No	Yes
Biscuits	2	Discretionary foods	No	Yes
Breadrolls, Baps & Bagels	1	Cereals/Grains	No	Yes
Breads	13	Cereals/Grains	No	Yes
Cakes	1	Discretionary foods	No	Yes
Canned Fatty Fish	4	Fish/Seafood	No	Yes
Canned Low Fat Fish	1	Fish/Seafood	No	Yes
Cereal Bars	2	Discretionary foods	No	Yes
Cereals	19	Cereals/Grains	No	Yes
Cheeses	12	Dairy/Alternatives	No	Yes
Chicken	4	Poultry	No	Yes
Chocolate	1	Discretionary foods	No	Yes
Chocolate Confectionery	1	Discretionary foods	No	Yes
Coffee	11	Tea/Coffee	No	Yes
Cows Milk	9	Dairy/Alternatives	No	Yes
Crackers & Crispbreads	4	Cereals/Grains ǀ Discretionary foods	Yes	Yes
Crisps	2	Discretionary foods	No	Yes
Dried Fruit	3	Fruit	No	Yes
Drinking Yogurts	1	Dairy/Alternatives	No	Yes
Duck	2	Poultry	No	Yes
Eggs	2	Eggs	No	Yes
Energy Drinks	1	Soft/energy/sports drinks	No	Yes
Fatty Fish	5	Fish/Seafood	No	Yes
Fish Products	5	Fish/Seafood	No	Yes
Flours	7	Cereals/Grains	No	Yes
Food Additives	3	Other/Non-pattern	No	No

<table>
<tr><th>Normalised source category</th><th>Unique FoodName entries, n</th><th>Final analytical food group(s)</th><th>FoodName-level split</th><th>PCA eligibility</th></tr>
<tr><td>Fruit Drinks</td><td>2</td><td>Soft/energy/sports drinks</td><td>No</td><td>Yes</td></tr>
<tr><td>Fruit Juices</td><td>8</td><td>Fruit | Mixed dishes/Condiments | Soft/energy/sports drinks | Vegetables</td><td>Yes</td><td>Mixed</td></tr>
<tr><td>Fruits</td><td>32</td><td>Fruit</td><td>No</td><td>Yes</td></tr>
<tr><td>Grains</td><td>7</td><td>Cereals/Grains</td><td>No</td><td>Yes</td></tr>
<tr><td>Herbs & Spices</td><td>3</td><td>Other/Non-pattern</td><td>No</td><td>No</td></tr>
<tr><td>Ice Creams</td><td>2</td><td>Discretionary foods</td><td>No</td><td>Yes</td></tr>
<tr><td>Lamb</td><td>2</td><td>Red/processed meat</td><td>No</td><td>Yes</td></tr>
<tr><td>Leafy Vegetables</td><td>8</td><td>Vegetables</td><td>No</td><td>Yes</td></tr>
<tr><td>Low Fat Fish</td><td>6</td><td>Fish/Seafood</td><td>No</td><td>Yes</td></tr>
<tr><td>Meat Dishes</td><td>16</td><td>Poultry | Red/processed meat</td><td>Yes</td><td>Yes</td></tr>
<tr><td>Meat Products</td><td>8</td><td>Poultry | Red/processed meat</td><td>Yes</td><td>Yes</td></tr>
<tr><td>Milkshakes & Smoothies</td><td>5</td><td>Dairy/Alternatives | Other/Non-pattern</td><td>Yes</td><td>Mixed</td></tr>
<tr><td>Miscellaneous</td><td>5</td><td>Discretionary foods | Mixed dishes/Condiments</td><td>Yes</td><td>Mixed</td></tr>
<tr><td>Mushrooms & Funghi</td><td>3</td><td>Vegetables</td><td>No</td><td>Yes</td></tr>
<tr><td>Non-Cows Milk</td><td>1</td><td>Dairy/Alternatives</td><td>No</td><td>Yes</td></tr>
<tr><td>Nuts & Seeds</td><td>15</td><td>Nuts/Seeds</td><td>No</td><td>Yes</td></tr>
<tr><td>Offal</td><td>2</td><td>Red/processed meat</td><td>No</td><td>Yes</td></tr>
<tr><td>Oils</td><td>5</td><td>Fats/Oils</td><td>No</td><td>Yes</td></tr>
<tr><td>Packet Soups</td><td>1</td><td>Mixed dishes/Condiments</td><td>No</td><td>No</td></tr>
<tr><td>Pastas</td><td>5</td><td>Cereals/Grains</td><td>No</td><td>Yes</td></tr>
<tr><td>Pastries & Buns</td><td>4</td><td>Cereals/Grains | Discretionary foods</td><td>Yes</td><td>Yes</td></tr>
<tr><td>Pickles & Chutney</td><td>2</td><td>Mixed dishes/Condiments</td><td>No</td><td>No</td></tr>
<tr><td>Pizzas</td><td>1</td><td>Cereals/Grains</td><td>No</td><td>Yes</td></tr>
<tr><td>Pork</td><td>7</td><td>Red/processed meat</td><td>No</td><td>Yes</td></tr>
<tr><td>Potatoes</td><td>3</td><td>Discretionary foods | Vegetables</td><td>Yes</td><td>Yes</td></tr>
<tr><td>Processed Milks & Powders</td><td>9</td><td>Dairy/Alternatives</td><td>No</td><td>Yes</td></tr>
<tr><td>Recipe</td><td>1</td><td>Cereals/Grains</td><td>No</td><td>Yes</td></tr>
<tr><td>Rice</td><td>4</td><td>Cereals/Grains</td><td>No</td><td>Yes</td></tr>
<tr><td>Roots | Tubers | Bulbs</td><td>17</td><td>Vegetables</td><td>No</td><td>Yes</td></tr>
</table>

Normalised source category	Unique *FoodName* entries, *n*	Final analytical food group(s)	*FoodName*-level split	PCA eligibility
Sauces	10	Mixed dishes/Condiments	No	No
Savouries	2	Cereals/Grains	No	Yes
Savoury Snacks	3	Discretionary foods	No	Yes
Seafood Dishes	2	Fish/Seafood	No	Yes
Shellfish	8	Fish/Seafood	No	Yes
Soft Drinks	10	Soft/energy/sports drinks	No	Yes
Soups	8	Mixed dishes/Condiments ǀ Pulses ǀ Vegetables	Yes	Mixed
Sports Drinks	3	Soft/energy/sports drinks	No	Yes
Spreading Fats	1	Fats/Oils	No	Yes
Squash & Cordials	5	Soft/energy/sports drinks	No	Yes
Sugars, Syrups & Preserves	6	Discretionary foods	No	Yes
Supplements	1	Other/Non-pattern	No	No
Sweets	1	Discretionary foods	No	Yes
Teas	8	Tea/Coffee	No	Yes
Turkey	2	Poultry	No	Yes
Vegetable Dishes	7	Discretionary foods ǀ Mixed dishes/Condiments ǀ Pulses ǀ Vegetables	Yes	Mixed
Vegetables-General	29	Vegetables	No	Yes
Water	6	Water	No	No
Yogurts	3	Dairy/Alternatives	No	Yes

FoodName-level split indicates that items within the same normalised source category were assigned to different analytical food groups according to the audited food-level mapping. *PCA eligibility* is reported as Yes when all listed analytical destinations were included in PCA, No when all were excluded, and Mixed when a split category contained both PCA-included and PCA-excluded destinations. Water, Mixed dishes/Condiments and Other/Non-pattern were excluded from PCA. The 77 normalised categories originated from 78 raw Category2 labels after resolving a trailing-space duplication in Fruit Juices. The category-level mapping summarised here is consistent with the final 18-group analytical structure and reproduces the 15 food-group totals used in the final PCA dataset.

Abbreviations: PCA, principal component analysis.

Classification notes: The source category “Recipe” corresponded to one distinct food entry labelled “Porridge Builder” in the original Nutritics database and was assigned to Cereals/Grains based on its product designation; the complete recipe composition was not independently available. The Pizzas category included “Pizza, cheese and tomato, retail”, which was retained in Cereals/Grains as part of the original analytical mapping. Categories such as Pastries & Buns were split at FoodName level when the distinction between staple cereal products and discretionary sweet products was relevant. The analytical food-group classification was developed for the present dietary-pattern analysis and should not be interpreted as a comprehensive nutritional classification of every individual food.

Table S2. Two-component principal component analysis: rotated food-group loadings, communalities, and variance explained.

Food group	Component 1	Component 2	Communality	Uniqueness
Alcoholic beverages	-0.280	-0.121	0.093	0.907
Cereals/Grains	**-0.729**	-0.228	0.583	0.417
Dairy/Alternatives	-0.180	**-0.532**	0.315	0.685
Discretionary foods	-0.369	-0.214	0.182	0.818
Eggs	-0.122	**-0.403**	0.177	0.823
Fats/Oils	-0.367	-0.388	0.285	0.715
Fish/Seafood	-0.259	**-0.482**	0.300	0.700
Fruit	**-0.525**	-0.284	0.356	0.644
Nuts/Seeds	**-0.636**	-0.052	0.407	0.593
Poultry	0.006	**-0.826**	0.682	0.318
Pulses	**-0.728**	0.114	0.543	0.457
Red/processed meat	-0.050	**-0.724**	0.527	0.473
Soft/energy/sports drinks	-0.069	**-0.516**	0.271	0.729
Tea/Coffee	-0.222	-0.125	0.065	0.935
Vegetables	**-0.707**	-0.018	0.500	0.500
**SS loadings**	**2.760**	**2.525**	—	—
**Variance explained (%)**	**18.40**	**16.84**	—	—
**Cumulative variance (%)**	**18.40**	**35.24**	—	—

Analyses were based on the 15 prespecified analytical food groups for 111 participants. Daily food-group intakes were transformed using log1p(g/day), standardised, and subjected to PCA followed by orthogonal varimax rotation with Kaiser normalisation. The signs of component loadings are mathematically arbitrary; the original two-component candidate orientation has been preserved and should not be equated with the sign or labels of the retained three-component solution.

Communality is the proportion of a food group’s variance accounted for by the two retained components; uniqueness = 1 − communality. Absolute loadings ≥ 0.40 are shown in bold. The two-component solution explained 35.24% of total variance (18.40% and 16.84%). The retained three-component solution explained 46.25%; its loadings and communalities are presented in main-text Table 3.

The parallel analysis of 1,000 simulated datasets supported retention of three components: the third observed eigenvalue was 1.651, exceeding its simulated 95th-percentile reference value of 1.484, whereas the fourth observed eigenvalue (1.192) did not exceed its corresponding reference value (1.367). The two-component solution is presented here as the prespecified candidate alternative, not as a replacement for the primary three-component analysis.

Table S3. PCA suitability, component-retention diagnostics and variance explained.

Diagnostic	Raw g/day	log1p(g/day)
**Overall KMO**	0.704	0.700
**Minimum individual KMO**	0.478	0.614
**Bartlett test *p*-value**	<0.001	<0.001
**Maximum absolute correlation**	0.500	0.610
**Components retained by parallel analysis**	1	3

Variance explained by the selected three-pattern solution

Pattern	SS loading	Variance (%)	Cumulative variance (%)
**Plant-rich**	2.445	16.30	16.30
**Animal-source foods**	2.278	15.18	31.48
**Energy-dense/discretionary**	2.214	14.76	46.25

KMO, Kaiser-Meyer-Olkin measure of sampling adequacy. The log1p-transformed scale was selected for the primary analysis to accommodate the marked right-skewness and structural zeros characteristic of several food-group distributions. This transformation also improved the minimum individual KMO. Analyses using the untransformed scale were retained as a sensitivity assessment. Component retention in the primary analysis was subsequently evaluated using parallel analysis, scree/eigenvalue inspection, interpretability and bootstrap stability.

Table S4. Adjusted sex comparisons across cohort characteristics, core diet, food groups and dietary-pattern scores.

Family	Outcome	Contrast	Adjusted difference (SD)	95% CI	*p*	BH-FDR *q*	Unadjusted Hedges *g*
Cohort anthropometry	Height	Male vs Female	+1.42	+1.12 to +1.72	<0.001	<0.001	+1.80
	Weight	Male vs Female	+0.49	+0.10 to +0.89	0.02	0.02	+0.52
	BMI	Male vs Female	-0.30	-0.72 to +0.11	0.15	0.17	-0.26
	Body fat	Male vs Female	-1.35	-1.68 to -1.03	<0.001	<0.001	-1.73
	Body water	Male vs Female	+1.32	+0.98 to +1.65	<0.001	<0.001	+1.62
	Muscle mass	Male vs Female	+1.62	+1.36 to +1.89	<0.001	<0.001	+2.47
	Bone mass	Male vs Female	+1.62	+1.35 to +1.89	<0.001	<0.001	+2.45
	Visceral fat index	Male vs Female	-0.08	-0.50 to +0.33	0.69	0.69	-0.04
	BMR	Male vs Female	+1.47	+1.18 to +1.77	<0.001	<0.001	+2.04
	Systolic BP	Male vs Female	+0.44	+0.04 to +0.84	0.03	0.04	+0.53
	Diastolic BP	Male vs Female	-0.37	-0.79 to +0.04	0.08	0.09	-0.34
	Pulse	Male vs Female	-0.56	-0.96 to -0.16	0.006	0.01	-0.52
Core diet	Energy intake	Male vs Female	+0.67	+0.28 to +1.06	0.001	0.005	+0.68
	Protein	Male vs Female	+0.04	-0.17 to +0.26	0.69	0.69	+0.62
	Total fat	Male vs Female	-0.24	-0.39 to -0.09	0.002	0.005	+0.41
	Carbohydrate	Male vs Female	+0.11	-0.03 to +0.25	0.12	0.15	+0.77
	Fibre	Male vs Female	-0.22	-0.45 to -0.00	0.047	0.08	+0.39
Food groups	Alcoholic beverages	Male vs Female	-0.64	-1.05 to -0.23	0.002	0.02	-0.37
	Cereals/Grains	Male vs Female	+0.20	-0.09 to +0.49	0.17	0.36	+0.73
	Dairy/Alternatives	Male vs Female	+0.07	-0.30 to +0.44	0.71	0.89	+0.35
	Discretionary foods	Male vs Female	-0.00	-0.37 to +0.37	>0.99	>0.99	+0.37
	Eggs	Male vs Female	-0.20	-0.63 to +0.24	0.37	0.56	-0.06
	Fats/Oils	Male vs Female	-0.59	-0.97 to -0.20	0.003	0.02	-0.22
	Fish/Seafood	Male vs Female	-0.15	-0.58 to +0.29	0.50	0.68	-0.01
	Fruit	Male vs Female	+0.06	-0.33 to +0.45	0.77	0.89	+0.37
	Nuts/Seeds	Male vs Female	-0.41	-0.81 to -0.01	0.04	0.16	-0.13

**Family**	**Outcome**	**Contrast**	**Adjusted difference (SD)**	**95% CI**	***p***	**BH-FDR *q***	**Unadjusted Hedges *g***
	Poultry	Male vs Female	+0.03	-0.37 to +0.43	0.87	0.94	+0.22
	Pulses	Male vs Female	-0.25	-0.63 to +0.13	0.19	0.36	+0.08
	Red/processed meat	Male vs Female	+0.18	-0.20 to +0.57	0.34	0.56	+0.41
	Soft/energy/sports drinks	Male vs Female	+0.37	-0.05 to +0.78	0.08	0.20	+0.58
	Tea/Coffee	Male vs Female	-0.43	-0.87 to +0.00	0.052	0.16	-0.25
	Vegetables	Male vs Female	-0.59	-0.95 to -0.23	0.002	0.02	-0.15
Dietary patterns	Plant-rich	Male vs Female	-0.43	-0.76 to -0.10	0.01	0.04	+0.01
	Animal-source foods	Male vs Female	+0.20	-0.19 to +0.60	0.31	0.31	+0.34
	Energy-dense/discretionary	Male vs Female	-0.20	-0.55 to +0.15	0.27	0.31	+0.24

Adjusted estimates are expressed in outcome SD units. Dietary outcomes other than total energy were adjusted for log-transformed total energy intake. BH-FDR correction was applied within prespecified outcome families. Anthropometric models were adjusted for age and geographical background. Total energy was adjusted for age and geographical background. All other dietary and dietary-pattern outcomes were additionally adjusted for log-transformed total energy intake.

Table S5. Adjusted global tests according to geographical background.

Family	Outcome	*n*	*F*	df	Partial eta^2	*p*	BH-FDR *q*
Cohort anthropometry	Height	109	9.56	2, 104	0.155	0.0002	0.002
	Weight	109	3.73	2, 104	0.067	0.03	0.07
	BMI	109	1.61	2, 104	0.030	0.21	0.27
	Body fat	109	1.06	2, 104	0.020	0.35	0.41
	Body water	109	2.02	2, 104	0.037	0.14	0.21
	Muscle mass	109	4.90	2, 104	0.086	0.009	0.04
	Bone mass	109	4.87	2, 104	0.086	0.01	0.04
	Visceral fat index	109	0.98	2, 104	0.018	0.38	0.41
	BMR	109	4.55	2, 104	0.081	0.01	0.04
	Systolic BP	109	2.01	2, 104	0.037	0.14	0.21
	Diastolic BP	109	0.17	2, 104	0.003	0.85	0.85
	Pulse	109	2.68	2, 104	0.049	0.07	0.15
Core diet	Energy intake	109	2.22	2, 104	0.041	0.11	0.14
	Protein	109	4.35	2, 103	0.078	0.02	0.04
	Total fat	109	3.99	2, 103	0.072	0.02	0.04
	Carbohydrate	109	1.47	2, 103	0.028	0.23	0.23
	Fibre	109	4.51	2, 103	0.081	0.01	0.04
Food groups	Alcoholic beverages	109	0.11	2, 103	0.002	0.90	0.96
	Cereals/Grains	109	0.62	2, 103	0.012	0.54	0.73
	Dairy/Alternatives	109	2.59	2, 103	0.048	0.08	0.24
	Discretionary foods	109	0.83	2, 103	0.016	0.44	0.69
	Eggs	109	0.93	2, 103	0.018	0.40	0.69
	Fats/Oils	109	2.80	2, 103	0.052	0.07	0.24
	Fish/Seafood	109	0.03	2, 103	0.001	0.97	0.97
	Fruit	109	0.15	2, 103	0.003	0.86	0.96
	Nuts/Seeds	109	2.10	2, 103	0.039	0.13	0.27
	Poultry	109	3.53	2, 103	0.064	0.03	0.17

Family	Outcome	*n*	*F*	df	Partial eta²	*p*	BH-FDR *q*
	Pulses	109	4.31	2, 103	0.077	0.02	0.12
	Red/processed meat	109	7.41	2, 103	0.126	0.001	0.02
	Soft/energy/sports drinks	109	0.79	2, 103	0.015	0.46	0.69
	Tea/Coffee	109	0.35	2, 103	0.007	0.70	0.88
	Vegetables	109	2.23	2, 103	0.042	0.11	0.27
Dietary patterns	Plant-rich	109	5.75	2, 103	0.101	0.004	0.006
	Animal-source foods	109	5.75	2, 103	0.100	0.004	0.006
	Energy-dense/discretionary	109	0.95	2, 103	0.018	0.39	0.39

Models were adjusted for sex and age; dietary outcomes other than total energy were additionally adjusted for log-transformed total energy intake. Formal geographical-background inference included Africa, Asia and Europe (*n*=109); Other (*n*=2) was excluded because of sparse cell size.

Table S6. Adjusted pairwise geographical-background comparisons.

Family	Outcome	Contrast	Adjusted difference (SD)	95% CI	*p*	BH-FDR *q*
Cohort anthropometry	Height	Africa vs Europe	-0.13	-0.48 to +0.22	0.46	0.55
		Asia vs Europe	-0.70	-1.06 to -0.34	0.0002	0.007
		Africa vs Asia	+0.57	+0.25 to +0.88	0.0005	0.009
	Weight	Africa vs Europe	-0.39	-0.86 to +0.09	0.11	0.25
		Asia vs Europe	-0.67	-1.15 to -0.18	0.007	0.045
		Africa vs Asia	+0.28	-0.14 to +0.71	0.19	0.36
	BMI	Africa vs Europe	-0.40	-0.90 to +0.10	0.11	0.25
		Asia vs Europe	-0.41	-0.92 to +0.09	0.11	0.25
		Africa vs Asia	+0.01	-0.43 to +0.45	0.96	0.96
	Body fat	Africa vs Europe	-0.05	-0.44 to +0.34	0.80	0.85
		Asia vs Europe	-0.26	-0.66 to +0.14	0.20	0.36
		Africa vs Asia	+0.21	-0.14 to +0.56	0.24	0.41
	Body water	Africa vs Europe	+0.22	-0.18 to +0.62	0.28	0.44
		Asia vs Europe	+0.41	+0.00 to +0.82	0.048	0.14
		Africa vs Asia	-0.19	-0.55 to +0.17	0.29	0.44
	Muscle mass	Africa vs Europe	-0.37	-0.68 to -0.05	0.02	0.11
		Asia vs Europe	-0.50	-0.83 to -0.18	0.003	0.02
		Africa vs Asia	+0.14	-0.15 to +0.42	0.34	0.46
	Bone mass	Africa vs Europe	-0.36	-0.68 to -0.04	0.03	0.11
		Asia vs Europe	-0.51	-0.84 to -0.18	0.003	0.02
		Africa vs Asia	+0.15	-0.13 to +0.44	0.30	0.44
	Visceral fat index	Africa vs Europe	-0.35	-0.84 to +0.15	0.17	0.36
		Asia vs Europe	-0.26	-0.77 to +0.25	0.31	0.45
		Africa vs Asia	-0.09	-0.53 to +0.36	0.70	0.79
	BMR	Africa vs Europe	-0.39	-0.74 to -0.04	0.03	0.11
		Asia vs Europe	-0.54	-0.89 to -0.18	0.004	0.03
		Africa vs Asia	+0.15	-0.16 to +0.46	0.35	0.47

Family	Outcome	Contrast	Adjusted difference (SD)	95% CI	*p*	BH-FDR *q*
	Systolic BP	Africa vs Europe	-0.46	-0.92 to -0.00	0.048	0.14
		Asia vs Europe	-0.31	-0.78 to +0.16	0.20	0.36
		Africa vs Asia	-0.16	-0.57 to +0.25	0.45	0.55
	Diastolic BP	Africa vs Europe	-0.08	-0.57 to +0.41	0.75	0.81
		Asia vs Europe	+0.04	-0.45 to +0.54	0.86	0.88
		Africa vs Asia	-0.12	-0.56 to +0.31	0.57	0.66
	Pulse	Africa vs Europe	+0.19	-0.29 to +0.66	0.43	0.55
		Asia vs Europe	+0.54	+0.05 to +1.02	0.03	0.11
		Africa vs Asia	-0.35	-0.78 to +0.07	0.11	0.25
Core diet	Energy intake	Africa vs Europe	+0.02	-0.45 to +0.49	0.94	0.94
		Asia vs Europe	+0.42	-0.06 to +0.90	0.09	0.18
		Africa vs Asia	-0.40	-0.82 to +0.02	0.06	0.18
	Protein	Africa vs Europe	-0.06	-0.30 to +0.18	0.62	0.72
		Asia vs Europe	-0.33	-0.58 to -0.08	0.01	0.050
		Africa vs Asia	+0.27	+0.05 to +0.49	0.02	0.06
	Total fat	Africa vs Europe	-0.14	-0.30 to +0.03	0.11	0.18
		Asia vs Europe	+0.08	-0.10 to +0.25	0.38	0.48
		Africa vs Asia	-0.21	-0.36 to -0.06	0.007	0.049
	Carbohydrate	Africa vs Europe	+0.14	-0.02 to +0.30	0.10	0.18
		Asia vs Europe	+0.11	-0.06 to +0.27	0.20	0.30
		Africa vs Asia	+0.03	-0.12 to +0.17	0.71	0.76
	Fibre	Africa vs Europe	-0.21	-0.46 to +0.04	0.10	0.18
		Asia vs Europe	+0.13	-0.13 to +0.38	0.33	0.45
		Africa vs Asia	-0.34	-0.56 to -0.11	0.004	0.049
Food groups	Alcoholic beverages	Africa vs Europe	-0.08	-0.55 to +0.38	0.72	0.91
		Asia vs Europe	-0.11	-0.59 to +0.37	0.66	0.91
		Africa vs Asia	+0.02	-0.40 to +0.45	0.92	0.96
	Cereals/Grains	Africa vs Europe	+0.09	-0.23 to +0.42	0.58	0.91

Family	Outcome	Contrast	Adjusted difference (SD)	95% CI	*p*	BH-FDR *q*
		Asia vs Europe	+0.19	-0.15 to +0.53	0.27	0.68
		Africa vs Asia	-0.10	-0.39 to +0.20	0.52	0.88
	Dairy/Alternatives	Africa vs Europe	-0.42	-0.79 to -0.05	0.03	0.21
		Asia vs Europe	-0.34	-0.72 to +0.05	0.09	0.36
		Africa vs Asia	-0.08	-0.42 to +0.26	0.63	0.91
	Discretionary foods	Africa vs Europe	+0.05	-0.37 to +0.47	0.81	0.94
		Asia vs Europe	+0.25	-0.18 to +0.69	0.25	0.68
		Africa vs Asia	-0.20	-0.58 to +0.18	0.30	0.68
	Eggs	Africa vs Europe	-0.10	-0.59 to +0.39	0.69	0.91
		Asia vs Europe	-0.33	-0.83 to +0.18	0.20	0.65
		Africa vs Asia	+0.23	-0.22 to +0.67	0.31	0.68
	Fats/Oils	Africa vs Europe	-0.22	-0.65 to +0.21	0.31	0.68
		Asia vs Europe	-0.52	-0.97 to -0.08	0.02	0.20
		Africa vs Asia	+0.30	-0.09 to +0.70	0.13	0.46
	Fish/Seafood	Africa vs Europe	-0.00	-0.49 to +0.48	0.99	0.99
		Asia vs Europe	-0.05	-0.55 to +0.45	0.84	0.94
		Africa vs Asia	+0.05	-0.39 to +0.49	0.82	0.94
	Fruit	Africa vs Europe	+0.08	-0.36 to +0.52	0.73	0.91
		Asia vs Europe	-0.03	-0.48 to +0.42	0.90	0.96
		Africa vs Asia	+0.11	-0.29 to +0.51	0.59	0.91
	Nuts/Seeds	Africa vs Europe	-0.19	-0.64 to +0.27	0.41	0.81
		Asia vs Europe	+0.24	-0.23 to +0.71	0.32	0.68
		Africa vs Asia	-0.43	-0.84 to -0.01	0.04	0.28
	Poultry	Africa vs Europe	+0.11	-0.33 to +0.55	0.63	0.91
		Asia vs Europe	-0.41	-0.87 to +0.04	0.08	0.34
		Africa vs Asia	+0.52	+0.12 to +0.93	0.01	0.13
	Pulses	Africa vs Europe	-0.15	-0.58 to +0.28	0.50	0.88
		Asia vs Europe	+0.42	-0.02 to +0.87	0.06	0.32

Family	Outcome	Contrast	Adjusted difference (SD)	95% CI	*p*	BH-FDR *q*
		Africa vs Asia	-0.57	-0.96 to -0.18	0.005	0.07
	Red/processed meat	Africa vs Europe	-0.04	-0.47 to +0.38	0.84	0.94
		Asia vs Europe	-0.72	-1.16 to -0.27	0.002	0.04
		Africa vs Asia	+0.67	+0.28 to +1.06	0.0009	0.04
	Soft/energy/sports drinks	Africa vs Europe	+0.01	-0.45 to +0.47	0.96	0.98
		Asia vs Europe	-0.23	-0.71 to +0.24	0.33	0.68
		Africa vs Asia	+0.25	-0.17 to +0.66	0.25	0.68
	Tea/Coffee	Africa vs Europe	-0.19	-0.69 to +0.30	0.45	0.84
		Asia vs Europe	-0.05	-0.56 to +0.46	0.86	0.94
		Africa vs Asia	-0.14	-0.59 to +0.31	0.53	0.88
	Vegetables	Africa vs Europe	-0.40	-0.81 to +0.01	0.06	0.32
		Asia vs Europe	-0.09	-0.52 to +0.33	0.66	0.91
		Africa vs Asia	-0.30	-0.68 to +0.07	0.11	0.42
Dietary patterns	Plant-rich	Africa vs Europe	-0.18	-0.55 to +0.19	0.34	0.51
		Asia vs Europe	+0.40	+0.01 to +0.78	0.045	0.10
		Africa vs Asia	-0.58	-0.92 to -0.24	0.001	0.01
	Animal-source foods	Africa vs Europe	+0.01	-0.41 to +0.43	0.96	0.96
		Asia vs Europe	-0.59	-1.02 to -0.15	0.009	0.03
		Africa vs Asia	+0.60	+0.22 to +0.98	0.002	0.01
	Energy-dense/discretionary	Africa vs Europe	-0.13	-0.53 to +0.26	0.50	0.57
		Asia vs Europe	-0.28	-0.69 to +0.13	0.18	0.32
		Africa vs Asia	+0.15	-0.21 to +0.51	0.42	0.54

Pairwise estimates are shown for transparency. Interpretation in the main manuscript should prioritize prespecified global tests and contrasts that survive BH-FDR correction, rather than nominal pairwise *p*-values.
